# Secretory factors from OP9 stromal cells delay differentiation and increase the expansion potential of adult erythroid cells *in vitro*

**DOI:** 10.1038/s41598-018-20491-1

**Published:** 2018-01-31

**Authors:** Kongtana Trakarnsanga, Marieangela C. Wilson, Kate J. Heesom, Tatyana N. Andrienko, Chatchawan Srisawat, Jan Frayne

**Affiliations:** 1 Department of Biochemistry, Faculty of Medicine Siriraj Hospital, Mahidol University, Bangkok, Thailand; 2 School of Biochemistry, Faculty of Biomedical Sciences, University of Bristol, Bristol, United Kingdom

## Abstract

Development of *in vitro* culture systems for the generation of red blood cells is a goal of scientists globally with the aim of producing clinical grade products for transfusion. Although mature reticulocytes can be efficiently generated by such systems, the numbers produced fall short of that required for therapeutics, due to limited proliferative capacity of the erythroblasts. To overcome this hurdle, approaches are required to increase the expansion potential of such culture systems. The OP9 mouse stromal cell line is known to promote haematopoietic differentiation of pluripotent stem cells, however an effect of OP9 cells on erythropoiesis has not been explored. In this study, we show not only OP9 co-culture, but factors secreted by OP9 cells in isolation increase the proliferative potential of adult erythroid cells by delaying differentiation and hence maintaining self-renewing cells for an extended duration. The number of reticulocytes obtained was increased by approximately 3.5-fold, bringing it closer to that required for a therapeutic product. To identify the factors responsible, we analysed the OP9 cell secretome using comparative proteomics, identifying 18 candidate proteins. These data reveal the potential to increase erythroid cell numbers from *in vitro* culture systems without the need for genetic manipulation or co-culture.

## Introduction

Development of *in vitro* culture systems for the generation of red blood cells has become a goal for scientists globally with the aim of producing clinical grade blood products for transfusion. Erythroid cells can be efficiently differentiated to reticulocytes from adult peripheral blood stem cells, however, extrapolated cell numbers fall short of the level required for therapeutics, due to limited proliferation capacity^1^. Strategies are therefore required to overcome this hurdle.

Macrophages are believed to facilitate erythroblast proliferation *in vivo*, with both adhesion molecules and secreted factors implicated (reviewed by Klei *et al*.^2^). Macrophages have also been shown to stimulate erythroid cell expansion *in vitro*, although direct contact with erythroid cells is required^3^. Addition of a cytoprotective polymer (poloxamer 188) to the culture medium of late stage erythroid cells has been shown to enhance survival of the cells and increase overall yield by 1.5 fold^4^. Other approaches, such as inhibition of FOXM1^5^ have also been shown to have, albeit a small positive effect on erythroid cell numbers. Hence identification of additional novel factors to increase expansion of erythroid cell numbers *in vitro* is required. Furthermore, as different factors may act on different pathways, combinatorial approaches utilising synergistic effects may enable greater expansion rates to be achieved.

The OP9 stromal cell line was established from a mouse with a M-CSF (macrophage colony stimulating factor) gene mutation resulting in lack of M-CSF production from this cell line^6^. Stromal cells producing M-CSF induce the differentiation of embryonic stem cells (ESC) down the monocyte-macrophage linage^6^. In contrast OP9 stromal cells lacking M-CSF promote differentiation down other haematopoietic linages (erythroid, myeloid and B- cell)^6–11^. Likewise, OP9 cells have been used in co-culture to support erythroid differentiation of pluripotent stem cells and to improve terminal differentiation^12,13^. In contrast adult peripheral blood stem cells undergo efficient erythroid differentiation *in vitro* without the need for support cells^14^. However, an effect of OP9 cells on the proliferation of adult erythroid cells has not previously been explored.

In this study we show that factors secreted by OP9 cells increase the proliferative capacity and hence yield of adult erythroid cultures, by delaying differentiation and hence maintaining self-renewing cells for an extended duration.

## Results

### Co-culture with OP9 cells increases proliferation potential of adult erythroid cell culture by delaying differentiation

To study the effect of OP9 cells on the proliferation potential of erythroblasts the cells were initially incubated under co-culture conditions. Adult peripheral blood CD34^+^ haematopoietic progenitors were isolated from leukocyte-reduction system cones obtained from healthy donors. Aliquots of 10^4^ CD34^+^ cells were seeded on a layer of confluent OP9 cells, or incubated without OP9 cells (control culture). The cells were cultured using the 3-stage erythroid culture system described by Griffith *et al*.^14^, with additional fetal bovine serum (10% rather than 2% v/v) to support the OP9 cells (no difference was observed between expansion or differentiation of erythroid cells cultured in 2% or 10% fetal bovine serum; data not shown). Cells were sequentially transferred between the primary, secondary and tertiary media of the culture system for an overall culture duration of 22 days. Cell number and viability was assessed throughout the culture. A sample of cells was also collected on day 20 of culture for morphological analysis.

**Figure 1:**
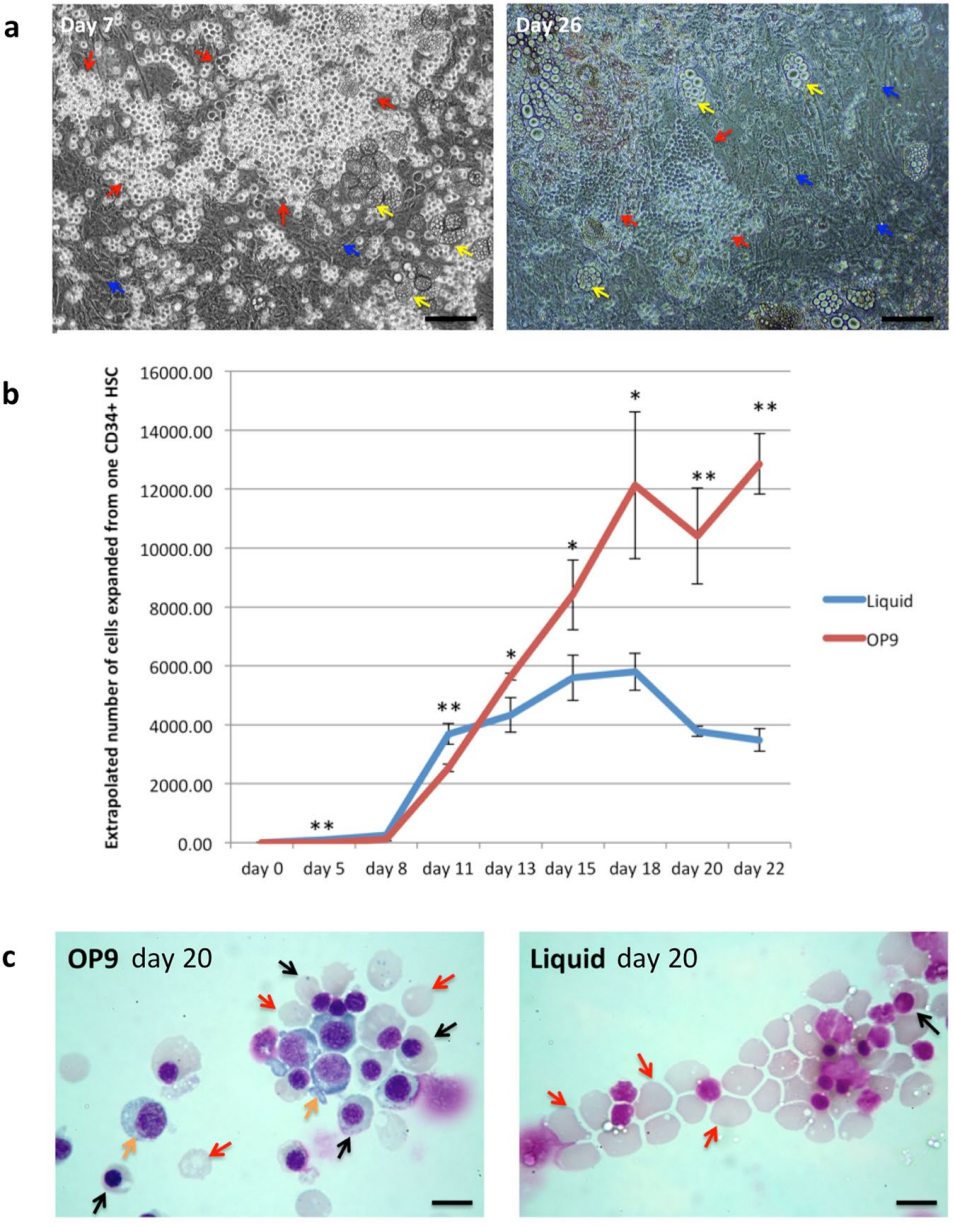
OP9 co-culture delays differentiation of erythroid cells. CD34^+^ cells were cultured with and without OP9 stromal cells in an erythroid differentiation culture. (**a**) Erythroblasts (red arrows) attached to OP9 stromal cells (blue arrows) at day 7 and day 26 of the co-culture taken under phase contrast microscopy (scale bars 100 μm). Yellow arrows indicate adipocytes. (**b**) Comparison of the number of erythroid cells from day 0 to day 22 in control liquid and OP9 co-culture (for OP9 co-culture, only detached cells being counted) (mean ± SD; n = 3, *p < 0.05, **p < 0.0; student’s t test). (**c**) Erythroid cells from OP9 co-culture and liquid culture at day 20 stained with Leishman reagent and analyzed by light microscopy (scale bars 10 μm). Orange arrows indicate polychromatic erythroblasts, black arrows indicate orthochromatic erythroblast, red arrows indicate reticulocytes.

Initially, during the first 11 days of culture, there appeared to be fewer cells in the OP9 co-culture compared to the control culture. However, visualization by phase contrast microscopy showed a substantial proportion of erythroblasts attached to the OP9 cells (Fig. 1a) which were excluded from the cell count, only detached cells being counted; the total cell number obtained is therefore an underestimate. Notwithstanding, from day 11 of culture a clear distinction between the cultures was apparent, with expansion rate of the control culture showing a marked decline (average 1.1-fold increase to day 18) whereas cell numbers in the OP9 co-culture continuing to double (Fig. 1b). Overall expansion of cell numbers in the OP9 co-culture was significantly higher than in the control culture, ~1.5 × 10^4^ compared to ~6 × 10^3^ respectively (n = 3; p < 0.001). There were still erythroblasts attached to the OP9 cells at the end of the culture, although substantially fewer than at early time points (Fig. 1a). Analysis of erythroid cell morphology on day 20 showed a significantly greater proportion of orthochromatic erythroblasts in the OP9 (57.2% ± 1.6%) compared to the control culture (8.6% ± 1.7%; n = 3, p < 0.001), along with 7.1% ± 1.7% polychromatic erythroblasts, whereas most cells in the control culture had already enucleated (Fig. 1c, Suppl Table 1), indicating delayed differentiation of a proportion of cells in the OP9 co-culture. To obtain morphological analysis at earlier time points the cultures were repeated. At day 14 almost 50% more pro-erythroblasts were detected in the OP9 compared to control culture. At day 18 a population of basophilic erythroblasts (8.6% ± 2.8%) was still present in the OP9 co-culture along with 19.3% ± 2.6% polychromatic erythroblasts, whereas all cells in the control culture had differentiated to orthochromatic erythroblasts or reticulocytes (Suppl Fig. 1; Table 1). Hence, the increased number of cells obtained in the OP9 co-cultures is due to persistence of earlier erythroblasts with proliferative potential to later stages of the culture compared to the control cultures.

**Table 1:** Morphological analysis of erythroid cells at different time points in the cultures with and without OP9 cells (mean ± SD, n = 3).

	Pro	Baso	Poly	Ortho	Retics
Liquid d8	70.8% ± 2.3%	27% ± 1.1%	2.2% ± 1.3%	0%	0%
OP9 d8	74.8% ± 2.5%	23.4% ± 1.5%	1.8% ± 1.0%	0%	0%
Liquid d14	8.6% ± 1.4%	11.7% ± 1.3%	23.5% ± 2.0%	33.4% ± 3.1%	22.8% ± 1.0%
OP9 d14	14.1% ± 1.6%	12.2% ± 2.9%	27.2% ± 1.9%	31.5% ± 2.9%	15.0% ± 0.6%
Liquid d18	0%	0%	0%	24.3% ± 4.4%	75.7% ± 4.4%
OP9 d18	0%	8.6% ± 2.8%	19.3% ± 2.6%	32.9% ± 1.8%	39.2% ± 4.4%

There was no obvious correlation between detachment of erythroblasts from the OP9 cells and erythroid maturation, cells at all stages of differentiation detected in the detached fraction (Suppl Fig. 1). A small proportion of OP9 cells differentiated to adipocyte-like cells as has been observed previously^15^, however the number did not increase notably during the cultures (Fig. 1a).

### Direct contact of erythroblasts with OP9 cells is not required to delay differentiation of erythroid cells

**Figure 2:**
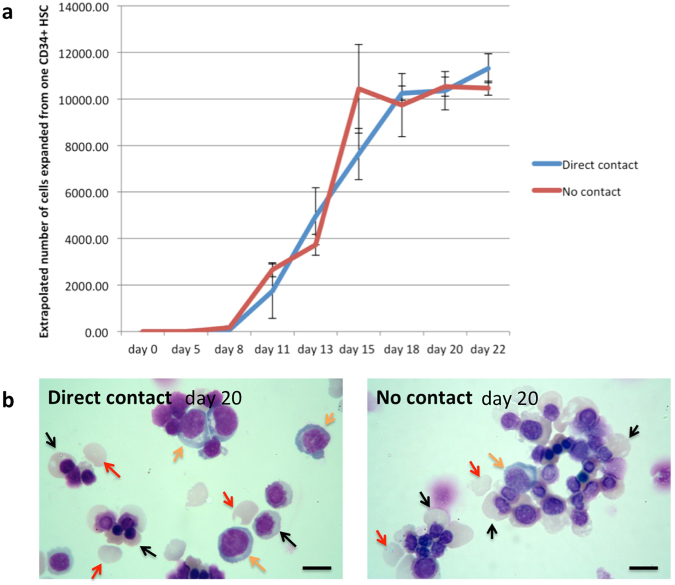
Direct contact of erythroblasts with OP9 cells is not required to delay differentiation of erythroid cells. CD34^+^ cells were maintained in OP9 co-culture with or without direct contact between the erythroid and OP9 cells (**a**) Comparison of the number of erythroid cells from day 0 to day 22 in OP9 co-culture with or without direct contact between the erythroid and OP9 cells (mean ± SD; n = 3, p > 0.05 at all time points; student’s t test). (**b**) Erythroid cells from OP9 co-culture with and without direct contact at day 20 stained with Leishman reagent and analyzed by light microscopy (scale bars 10 μm). Orange arrows indicate polychromatic erythroblasts, black arrows indicate orthochromatic erythroblast, red arrows indicate reticulocytes.

To determine whether direct contact of erythroblasts with OP9 cells is required, OP9 cells were seeded and maintained in wells of a tissue culture plate until confluent. Aliquots of 2,500 CD34^+^ progenitors were either seeded directly onto the OP9 cells or onto a NUNC tissue culture insert within the well of a tissue culture plate, preventing direct contact between the OP9 and CD34^+^ cells. The co-cultures were performed using the 3-stage erythroid culture system as for the previous experiment. The expansion profile for erythroblasts in direct contact with OP9 cells was the same as in experiment 1 above. However, unlike the control culture, in which the expansion rate declined from day 11, the number of erythroid cells in cultures with indirect exposure to OP9 cells continued to increase, paralleling the profile of the cultures with direct contact (Fig. 2a). There was no difference in the number of cells from this time point onwards between the two groups, or in the overall expansion of cell numbers. The morphology of cells on day 20 was also similar between the groups (Fig. 2b; Suppl Table 2). Hence direct contact between the OP9 cells and erythroblasts is not required to delay differentiation of erythroblasts.

**Table 2:** Morphological analysis of day 19 erythroid cells maintained in OP9 conditioned media and control media (mean ± SD, n = 3, student’s t-test).

	OP9 conditioned media	control media	p value
Orthochromatic	9.3% ± 1.1%	9.6% ± 1.7%	0.825976502
Reticulocyte	90.7% ± 1.1%	90.4% ± 1.7%	0.825976502

### Conditioned medium from OP9 cells delays differentiation and facilitates prolonged expansion of erythroblasts

**Figure 3:**
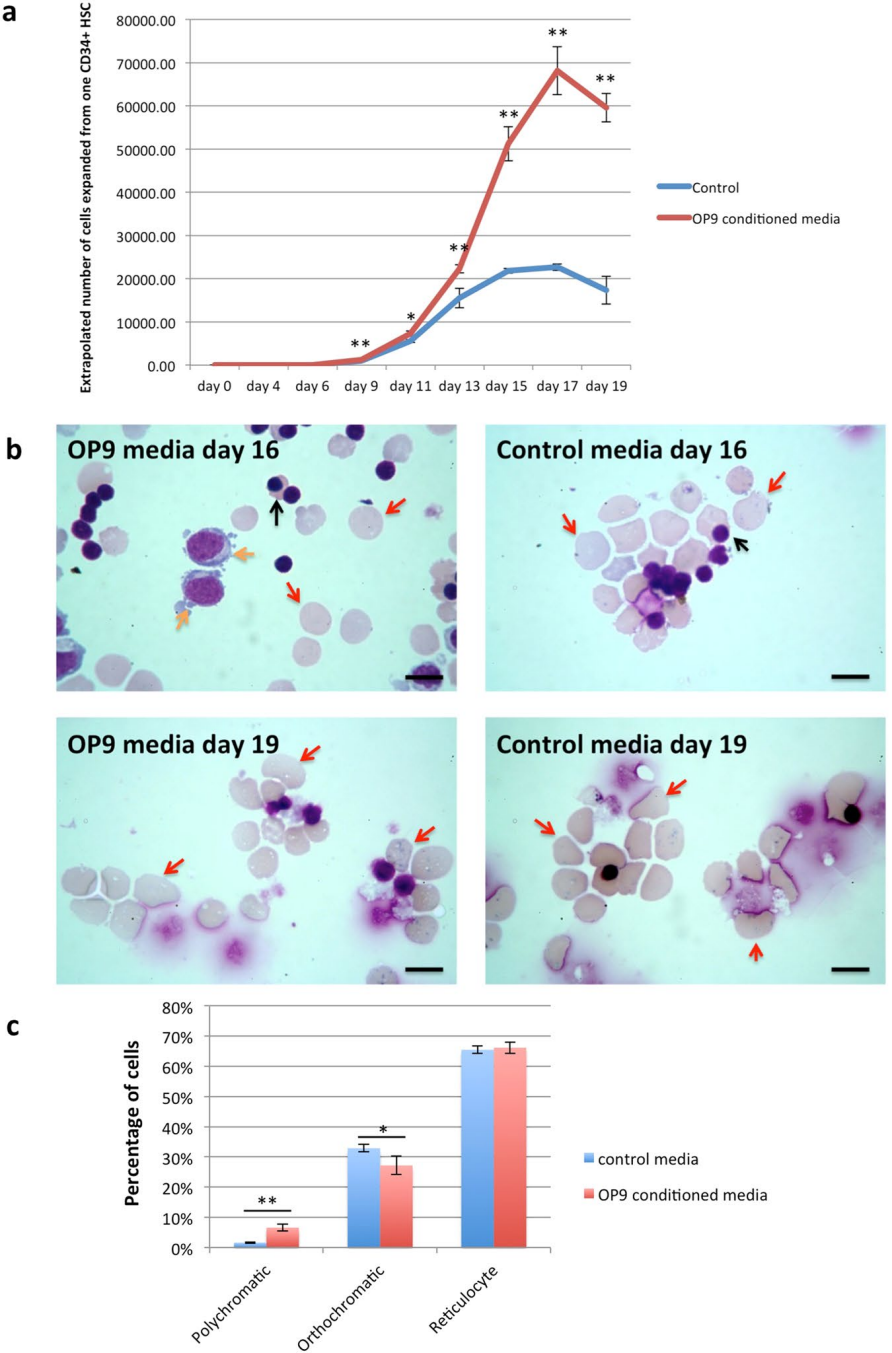
OP9-conditioned culture media delays differentiation of erythroid cells and increases expansion potential of cultures. CD34^+^ cells were cultured with OP9-conditioned culture media or control culture media for 19 days. (**a**) Comparison of the number of erythroid cells from day 0 to day 19 in cultures with OP9-conditioned media or control media (mean ± SD; n = 3, *p < 0.05, **p < 0.01; student’s t test). (**b**) Erythroid cells maintained in OP9-conditioned culture media or control culture media at day 16 and 19 stained with Leishman reagent and analyzed by light microscopy (scale bars 10 μm). Orange arrows indicate polychromatic erythroblasts, black arrows indicate orthochromatic erythroblast, red arrows indicate reticulocytes. (**c**) Morphological analysis of day 16 erythroid cells maintained in OP9 conditioned media and control media (mean ± SD; n = 3, *p < 0.05, **p < 0.01; student’s t test).

The use of co-culture, particularly inclusion of cells of non-human origin, is both in-practical and undesirable when considering therapeutic development. Our next step was therefore to determine if conditioned media from OP9 cells was adequate to obtain the observed effects. OP9 cells were incubated in primary, secondary and tertiary media for 48 hours, the media collected and filtered to remove any contaminating cells. Aliquots of 10^4^ CD34^+^ cells were incubated in these media or in the normal 3-stage media. Cells were counted throughout the culture. The number of erythroid cells in cultures with OP9-conditioned media was significantly higher at all time points from day 9 onwards than in control cultures (Fig. 3a). Overall expansion of cell numbers in the OP9-conditioned media was also significantly higher than in the control media, ~7 × 10^4^ compared to ~2 × 10^4^ respectively (n = 3; p < 0.001). Since this experiment was performed using only liquid culture conditions the number of cells could be maintained at optimal concentrations for each stage^14^ which results in higher numbers of cells in the control group than in previous experiments. On day 16, cultures with OP9-conditioned media had significantly more polychromatic erythroblasts still present (6.6% ± 1.2%) than control cultures (1.6% ± 0.2%; n = 3, p < 0.001) (Fig. 3b,c). Coupled with the greater overall number of cells obtained with OP9-conditioned media, the data indicate factors secreted by the OP9 cells delay differentiation and facilitate prolonged expansion of earlier erythroid cell populations. At later time points, the cultures were more synchronous than in the previous experiments, with similar proportions of orthochromatic erythroblasts and reticulocytes (Table 2), however the total number of these cell types were significantly higher in the OP9-conditioned media; at day 19, 5.57 × 10^7^ ± 8 × 10^6^ orthochromatics* and 5.4 × 10^8^ ± 2.7 × 10^7^ reticulocytes** compared to 1.66 × 10^7^ ± 3.63 × 10^6^ orthochromatics and 1.57 × 10^8^ ± 2.98 × 10^7^ reticulocytes respectively in the control media (n = 3, *p = 0.0015, **p < 0.001).

### Identification of OP9 secretory proteins using isobaric Tandem Mass Tag labelling and mass spectrometry

Finally, to identify factors in the OP9-conditioned media that may act on the erythroblasts to delay differentiation, isobaric Tandem Mass Tag labelling and nanoLC-MS/MS was used^11^ to compare the abundance of proteins in the OP9-conditioned and normal (control) media (see Methods). The raw data files were processed and quantified using Proteome Discoverer software v2.1 (Thermo Scientific) and searched against the UniProt Mouse (78740 entries) database using the SEQUEST algorithm to identify proteins secreted by the murine OP9 cells. For analysis only quantifications obtained from two or more unique peptides per protein were considered, with a comparative threshold of 2^16^. Using these criteria, 90 proteins were quantified (Suppl Table 3A). As cells with self-renewal capacity were detected in all 3 media of the culture system, we screened the data for proteins that were consistently at a higher level across all three OP9-conditioned media compared to respective control media, identifying 18 proteins (Table 3). No proteins were at a higher level across the control media. However, to confirm that the proteins identified at a higher level in the OP9-conditioned media originated from the OP9 cells, and not from the serum components of the media with variation in level introduced during preparation, we next searched the raw data files against the UniProt Human (134169 entries) and Bovine (31855 entries) databases (Suppl Tables 3B and C). Again, no proteins were consistently detected at a higher level across the control media. However, 7 proteins at a higher level across all OP9 media were detected on screening the bovine database and 8 on screening the human database, 11 of which were common to those identified at a higher level across OP9 media from the mouse database search. We therefore interrogated the data for each individual peptide to identify the source of the proteins. Peptides with sequences specific to just the bovine and/or human orthologue of a protein, were not at a higher level across the OP9 conditioned media, only peptides with a sequence also common to the mouse being higher. In contrast 15 of the proteins detected at a higher level across all OP9 conditioned media from the mouse database search had mouse specific peptides which were at a higher level (Suppl Table 4). For the remaining 3 proteins all peptides detected were common to the mouse and bovine and/or human orthologues. Thus, the proteins detected at a higher level from the bovine and human database searches were quantified only from peptides common to the respective mouse orthologue. The analyses confirm that all 18 candidate proteins identified were secreted by the OP9 cells and not of human or bovine origin.

**Table 3:** Proteins consistently presented at a higher level across OP9-conditioned primary, secondary and tertiary media compared to respective control media.

Accession	Description	Unique Peptides	Peptides	Primary OP9/Ctrl	Secondary OP9/Ctrl	Tertiary OP9/Ctrl
Q3TXQ4	Epididymal secretory protein E1 GN = Npc2	2	2	**39.939**	**60.295**	**44.861**
Q62356	Follistatin-related protein 1 GN = Fstl1	2	2	**21.167**	**19.100**	**9.835**
P11859	Angiotensinogen GN = Agt	5	5	**9.024**	**20.481**	**9.918**
Q5NCU4	SPARC GN = Sparc	11	11	**6.504**	**12.783**	**7.595**
Q3TP88	Collagen Type I Alpha 2 Chain GN = Col1a2	2	2	**5.488**	**6.549**	**3.497**
Q9QUN9	Dickkopf-related protein 3 GN = Dkk3	4	4	**4.486**	**9.849**	**5.371**
Q02819	Nucleobindin-1 GN = Nucb1	9	9	**4.225**	**17.923**	**5.085**
A0A0A6YWC8	Vimentin GN = Vim	3	3	**4.065**	**6.724**	**2.217**
P10605	Cathepsin B GN = Ctsb	9	9	**3.667**	**7.885**	**5.122**
P61982	14–3–3 protein gamma GN = Ywhag	3	5	**3.372**	**3.440**	**5.582**
Q9WUU7	Cathepsin Z GN = Ctsz	5	5	**3.030**	**8.107**	**2.966**
Q60994	Adiponectin GN = Adipoq	5	5	**2.756**	**16.040**	**3.943**
P11087	Collagen alpha-1(I) chain GN = Col1a1	4	4	**2.679**	**3.763**	**2.071**
Q8K173	Col3a1 protein GN = Col3a1	2	2	**2.660**	**3.384**	**2.419**
P03953	Complement factor D GN = Cfd	3	3	**2.481**	**12.126**	**7.427**
O09164	Extracellular superoxide dismutase [Cu-Zn] GN = Sod3	2	2	**2.340**	**6.509**	**5.747**
H3BLB7	Insulin-like growth factor-binding protein 4 GN = Igfbp4	2	2	**2.102**	**12.270**	**3.305**
P16015	Carbonic anhydrase 3 GN = Ca3	2	2	**2.058**	**6.284**	**3.168**

Culture media were serum depleted, fractionated and proteins subjected to trypsin digest, with resultant peptides labelled with TMTs for nanoLC-MS/MS based quantitation. Values show the ratio of protein levels between OP9-conditioned media and control media. Proteins were quantified from at least two unique peptides. Peptides and unique peptides; the total number of peptide sequences and number of unique peptides identified for that protein. Proteome Discoverer software v1.4 was used for analysis.

## Discussion

This study shows that co-culture of adult peripheral blood CD34^+^ haematopoietic stem cells with OP9 stromal cells maintains a population of self-renewing erythroblasts for a prolonged period, resulting in an increased cell yield per culture. Moreover, direct contact between OP9 cells and erythroblasts is not required, the same effect being achieved even with OP9 conditioned media; the number of reticulocytes obtained consistently around 3.5 fold higher than in control cultures.

Previous studies have utilized co-culture with macrophages to enhance *in vitro* erythropoiesis, based on the premise that *in vivo* erythropoiesis occurs in erythroblastic islands supported by a central macrophage. Such macrophages are surrounded by various stages of developing erythroid cells, from CFU-E to reticulocytes^17^, and are believed to be important for supporting erythroblast proliferation and differentiation^17^. However, macrophages are clearly not essential *in vitro* as erythroid cells can be successfully differentiated from CD34^+^ cells in isolation with high enucleation rates^14^. Notwithstanding, macrophages may further enhance erythropoietic culture systems, as co-culture of human erythroblasts with macrophages has been shown to increase expansion rates by a similar magnitude to that in our present study. However, direct contact of erythroid cells with macrophages was required to achieve the effect^3^, which is undesirable when considering development for therapeutics due to potential contamination of the product with nucleated cells, and also the requirement for immune compatibility between macrophage and erythroid cells. In contrast our study shows not only OP9 co-culture, but also the application of just factors secreted by OP9 cells delay differentiation and facilitate prolonged expansion of earlier erythroid cell populations, importantly with no downstream block to terminal differentiation or enucleation. It is therefore likely that the active factors secreted by OP9 cells are distinct to those expressed by macrophages, and present a potentially novel way to increase erythroid cell numbers without the need for genetic manipulation or co-culture.

Currently, with the culture system used in our study >10^5^ fold expansion of erythroid cells can be achieved in larger scale cultures^18^. As approximately 10^6^ adult stem cells are isolated from an apheresis cone, this gives a yield of around 10^11^ erythroid cells from a single donor. By extrapolation, OP9-conditioned media would increase this yield to around 3.5 × 10^11^. Enucleation rates *in vitro* vary between 60–95%^14,18^ for cells from different donors, which therefore gives a final potential yield of 2–3 × 10^11^ reticulocytes per culture. 1 unit of blood contains around 2 × 10^12^ RBCs. However, the age of the population is asynchronous with a proportion of cells immediately removed from circulation following transfusion as are at end of lifespan. In contrast all cells from *in vitro* cultures are nascent, and therefore closely synchronous in age, with the number of cells required for transfusion anticipated to be less than from donor blood. Thus the number of cells achieved from *in vitro* cultures is now approaching that required for a therapeutic product. Notwithstanding, further improvement is still required including development of custom filters as presently a proportion of reticulocytes are lost during this final process, and transfer of the culture system to GMP conditions.

As a preliminary approach to identify the factors responsible for the delayed differentiation in our study, we analysed the secretome of the OP9 cells. Of the 18 candidate proteins identified, a role in promoting erythroid cell proliferation has previously been reported for angiotensinogen^19^, in promoting HSC proliferation for NPC2^20^, 14–3–3 gamma^21^ and adiponectin^22^, and in promoting proliferation of other cells types for follistatin-related protein 1^23^, DKK^24^, secreted vimentin^25^, cathepsin B^26^ and extracellular superoxide dismutase^27^. Further studies are now underway to generate recombinant protein for each of the identified proteins to investigate their effect on erythroid cell expansion, both singularly and in various combinations to determine whether an individual factor or synergy between several factors is required.

## Materials and Methods

### Isolation of adult CD34+ cells

Leucocyte reduction system (LRS) cones were obtained from healthy donors with written informed consent for research use in accordance with the Declaration of Helsinki and approved by Siriraj Institutional Review Board (COA no. Si019/2016). Three LRS cones from 3 donors were used for each part of experiment (n = 3). The CD34^+^ cells were isolated from the peripheral blood mononuclear cell fraction using a MiniMacs direct CD34^+^ progenitor cell isolation kit following the manufacturer’s instructions.

### Erythroid differentiation of CD34+ cells

The CD34+ cells were cultured using the 3-stage erythroid culture system. During the first 8 days the cells were maintained in Basic medium which was Iscove’s medium (Biochrom) containing 3% (v/v) human AB serum (Sigma-Aldrich), 10% fetal calf serum (Hyclone, Fisher Scientific, Ltd), 10 μg/ml insulin (Sigma-Aldrich), 3 U/ml heparin (Sigma-Aldrich), 3 U/ml EPO (Roche), 200 μg/ml transferrin (R&D Systems) and 1 U/ml penicillin/streptomycin (Sigma-Aldrich) supplemented with 10 ng/ml SCF (R&D Systems) and 1 ng/ml IL-3 (R&D Systems) (primary medium). IL-3 and SCF were withdrawn from the medium on day 8 (secondary medium) and 11 (tertiary medium), respectively. In addition, extra transferrin was added to the medium to the final concentration of 500 μg/ml from day 11 onward. The cells were counted and medium was added every other day. The cultured cells were maintained at 37 °C, 5% CO_2_ throughout the culture period. At indicated time points, aliquots of cells were collected for morphological analysis using cytospin and Leishman staining. Two sample equal variance t-test was carried out to determine the statistic significances of cell numbers and cell types.

### Culture system for OP9 cells

OP9 mouse stromal cells were maintained in the OP9 growth medium, consisting of αMEM culture medium (Invitrogen) containing 100 μM 1-Thioglycerol (MTG) (Sigma-Aldrich), 100 U/ml penicillin/streptomycin and 20% (v/v) FCS. OP9 cells were separated and expanded every 4 days, when the cells were confluent, by trypsinisation. The harvested cells were seeded onto a 10-cm dish coated with 0.1% bovine gelatin solution (Sigma-Aldrich).

### Tandem Mass Tag labeling, preparation of samples for Mass Spectrometry, database search parameters and acceptance criteria for identifications

As all media contain both bovine and human serum, the high level of proteins from which could mask identification of proteins secreted by the OP9 cells, the media were serum depleted using a Pierce albumin depletion kit for bovine proteins followed by Pierce top 12 abundant protein depletion spin columns for human proteins. Remaining proteins were subject to trypsin cleavage, and the resultant peptides labeled with isobaric Tandem Mass Tags for comparative quantitation, and analysed by nanoLC-MS/MS, as previously described^11^. Following analysis against the UniProt Mouse database (see main text) we next searched the raw data files against the UniProt Human (134169 entries) and Bovine (31855 entries) databases (Suppl Table 4). For analysis only quantifications obtained from two or more unique peptides per protein were considered, with a comparative threshold of 2^16^.

## Electronic supplementary material

Supplementary Information
