# Supplementary material for: Secretory factors from OP9 stromal cells delay differentiation and increase the expansion potential of adult erythroid cells in vitro
Source: Sci Rep. 2018 Jan 31;8:1983. doi: 10.1038/s41598-018-20491-1 (PMC5792592; doi:10.1038/s41598-018-20491-1)
Supplement: Supplementary file 1 — Supplementary Information [file 41598_2018_20491_MOESM1_ESM.pdf]

Secretory factors from OP9 stromal cells delay differentiation and increase the expansion potential of adult erythroid cells *in vitro*

Kongtana Trakarnsanga[1,2], Marieangela C. Wilson[2], Kate J. Heesom[2], Tatyana N. Andrienko[2], Chatchawan Srisawat[1] and Jan Frayne[2*].

[1]Department of Biochemistry, Faculty of Medicine Siriraj Hospital, Mahidol University, Bangkok, Thailand; [2]School of Biochemistry, Faculty of Biomedical Sciences, University of Bristol, United Kingdom.

* Corresponding author: Jan.Frayne@bristol.ac.uk

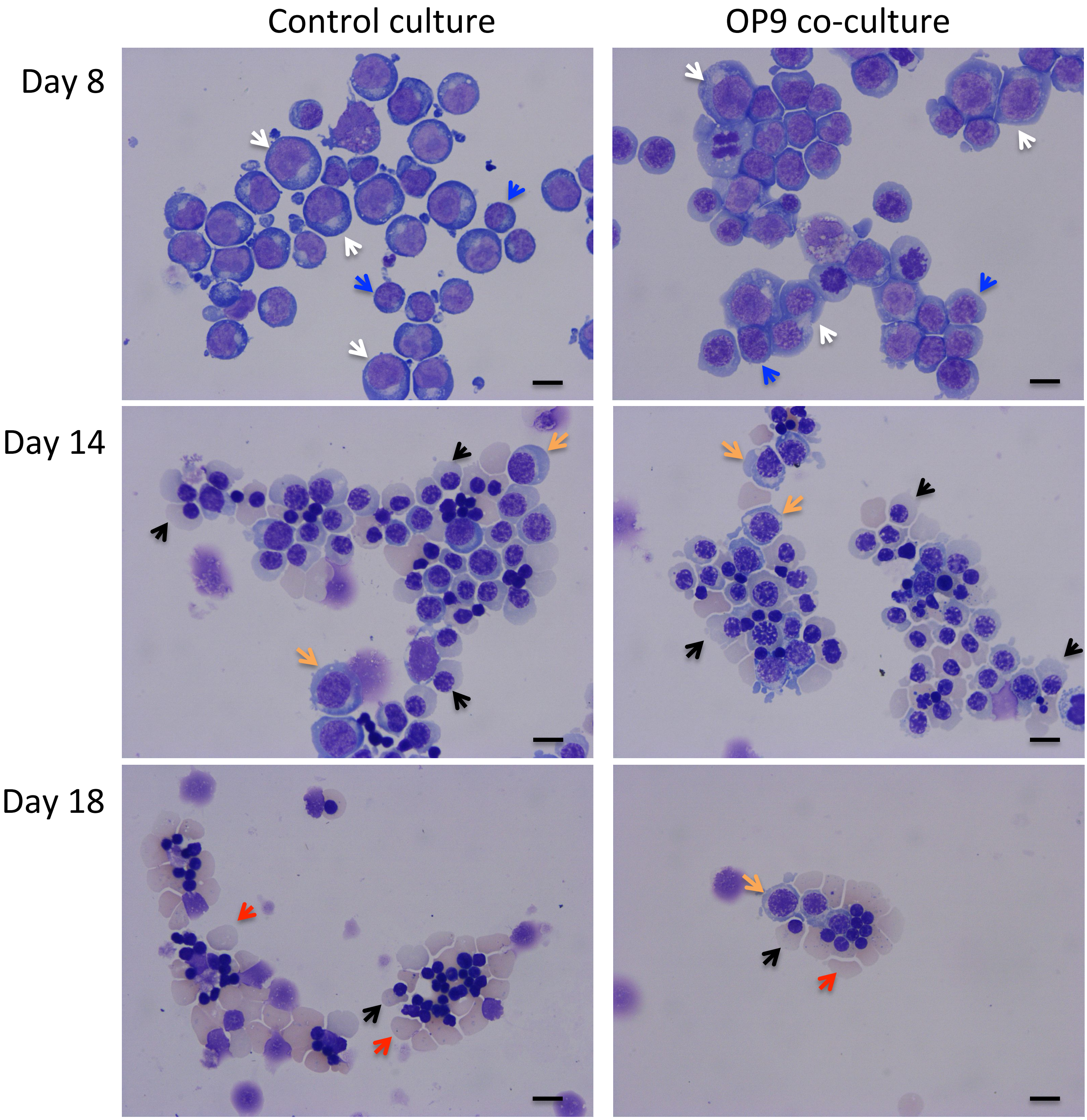

Supplementary Figure 1 OP9 co-culture delays differentiation of erythroid cells. CD34+ cells were cultured with and without OP9 stromal cell in erythroid differentiation culture. Erythroid cells from OP9 co-culture and liquid culture at day 8, 14 and 18 stained with Leishman reagent and analyzed by light microscopy (scale bars = 10 μm). white arrows = proerythroblast, blue arrows = basophillic erythroblasts, orange arrows = polychromatic erythroblasts, black arrows = orthochromatic erythroblast, red arrows = reticulocytes.

Supplementary Table 1 Morphological analysis of day 20 erythroid cells cultured with and without OP9 cells (mean ± SD, n=3, student's t-test).

	Liquid culture	OP9 co-culture	p value
Polychromatic	0%	7.1%±1.7%	0.0021866491
Orthochromatic	8.6%±1.7%	57.2%±1.6%	0.0000035131
Reticulocyte	91.4%±1.7%	35.7%±1.0%	0.0000011105

Supplementary Table 2 Morphological analysis of day 20 erythroid cells from OP9 co-culture with and without direct contact (mean ± SD, n=3, student's t-test).

	Direct contact	No contact	p value
Polychromatic	4.8%±0.6%	4.5%±0.9%	0.588142079
Orthochromatic	53.3%±3.3%	55.3%±3.5%	0.524483989
Reticulocyte	41.8%±2.9%	40.3%±3.1%	0.553709721

Supplementary Table 3. Details of proteins identified by MS in OP9-conditioned and control media. Analysis was performed against the UniProt Mouse (3A), Human (3B) and Bovine (3C) database.

Supplementary Table 3A

Accession	Description	Coverage	Unique Peptides	Peptides	PSMs	P OP9/Ctrl	S OP9/Ctrl	T OP9/Ctrl
P62259	14-3-3 protein epsilon OS=Mus musculus GN=Ywhae PE=1 SV=1 - [1433E_MOUSE]	16.08	2	4	4	1.683	**3.202**	**2.171**
P61982	14-3-3 protein gamma OS=Mus musculus GN=Ywhag PE=1 SV=2 - [1433G_MOUSE]	18.22	3	5	5	**3.372**	**3.440**	**5.582**
Q5MPX5	Activated leukocyte cell adhesion molecule soluble isoform OS=Mus musculus GN=Alcam PE=1 SV=1 - [Q5MPX5_MO	18.80	2	2	2	0.794	1.835	1.103
Q60994	Adiponectin OS=Mus musculus GN=Adipoq PE=1 SV=2 - [ADIPO_MOUSE]	24.70	5	5	8	**2.756**	**16.040**	**3.943**
Q61282	Aggrecan core protein OS=Mus musculus GN=Acan PE=1 SV=2 - [PGCA_MOUSE]	1.45	3	3	3	0.883	1.758	1.036
Q6GQT1	Alpha-2-macroglobulin-P OS=Mus musculus GN=A2m PE=2 SV=2 - [A2MG_MOUSE]	3.12	4	4	7	**0.498**	1.368	1.385
P97449	Aminopeptidase N OS=Mus musculus GN=Anpep PE=1 SV=4 - [AMPN_MOUSE]	4.45	5	5	5	0.716	**2.597**	1.255
Q99KE6	Amy1 protein (Fragment) OS=Mus musculus GN=Amy1 PE=2 SV=1 - [Q99KE6_MOUSE]	8.67	2	2	3	1.727	**4.522**	**2.346**
P11859	Angiotensinogen OS=Mus musculus GN=Agt PE=1 SV=1 - [ANGT_MOUSE]	16.56	5	5	6	**9.024**	**20.481**	**9.918**
A0A097PUD0	Anti-dectin-1 15E2 light chain OS=Mus musculus PE=2 SV=1 - [A0A097PUD0_MOUSE]	36.17	5	5	14	**0.420**	1.169	**2.885**
A0A097PUG1	Anti-lox-1 15C4 heavy chain OS=Mus musculus PE=2 SV=1 - [A0A097PUG1_MOUSE]	21.37	7	7	13	**0.430**	1.211	**2.284**
P32261	Antithrombin-III OS=Mus musculus GN=Serpinc1 PE=1 SV=1 - [ANT3_MOUSE]	28.39	12	12	41	0.612	1.843	1.150
P05201	Aspartate aminotransferase, cytoplasmic OS=Mus musculus GN=Got1 PE=1 SV=3 - [AATC_MOUSE]	5.08	2	2	2	0.808	**3.594**	1.409
B9EIU3	Attractin OS=Mus musculus GN=Atrn PE=2 SV=1 - [B9EIU3_MOUSE]	9.59	12	12	23	0.792	**2.203**	1.207
Q8CIF4	Biotinidase OS=Mus musculus GN=Btd PE=1 SV=2 - [BTD_MOUSE]	4.62	2	2	3	0.861	2.278	1.592
Q8R016	Bleomycin hydrolase OS=Mus musculus GN=Blmh PE=1 SV=1 - [BLMH_MOUSE]	7.91	3	3	4	0.691	**2.159**	1.252
Q8VDK4	Cadherin 13 OS=Mus musculus GN=Cdh13 PE=2 SV=1 - [Q8VDK4_MOUSE]	10.92	5	5	5	1.199	**2.063**	0.947
P62204	Calmodulin OS=Mus musculus GN=Calm1 PE=1 SV=2 - [CALM_MOUSE]	18.79	3	3	3	1.714	**2.567**	1.761
G3UWV3	Calumenin OS=Mus musculus GN=Calu PE=1 SV=1 - [G3UWV3_MOUSE]	27.27	3	3	3	1.621	**17.907**	**9.115**
P16015	Carbonic anhydrase 3 OS=Mus musculus GN=Ca3 PE=1 SV=3 - [CAH3_MOUSE]	6.92	2	2	2	**2.058**	**6.284**	**3.168**
Q9WVJ3	Carboxypeptidase Q OS=Mus musculus GN=Cpq PE=1 SV=1 - [CBPQ_MOUSE]	5.74	2	2	2	0.728	**2.250**	1.307
Q8R555	Cartilage acidic protein 1 OS=Mus musculus GN=Crtac1 PE=2 SV=1 - [CRAC1_MOUSE]	4.80	2	2	2	0.955	**2.217**	1.620
P10605	Cathepsin B OS=Mus musculus GN=Ctsb PE=1 SV=2 - [CATB_MOUSE]	30.68	9	9	11	**3.667**	**7.885**	**5.122**
F8WIR1	Cathepsin D OS=Mus musculus GN=Ctsd PE=1 SV=1 - [F8WIR1_MOUSE]	9.49	2	2	3	1.609	**2.402**	1.699
Q9WUU7	Cathepsin Z OS=Mus musculus GN=Ctsz PE=1 SV=1 - [CATZ_MOUSE]	17.32	5	5	5	**3.030**	**8.107**	**2.966**
E0CY16	Cell adhesion molecule 1 OS=Mus musculus GN=Cadm1 PE=1 SV=1 - [E0CY16_MOUSE]	17.18	2	5	5	0.974	1.547	1.300
G3X9T8	Ceruloplasmin OS=Mus musculus GN=Cp PE=1 SV=1 - [G3X9T8_MOUSE]	6.98	6	6	50	0.674	**2.216**	1.210
P03953	Cfd protein OS=Mus musculus GN=Cfd PE=2 SV=1 - [B7ZNS9_MOUSE]	18.99	3	3	3	**2.481**	**12.126**	**7.427**
Q03311	Cholinesterase OS=Mus musculus GN=Bche PE=1 SV=2 - [CHLE_MOUSE]	4.31	2	2	2	0.749	1.602	1.227
Q8K173	Col3a1 protein (Fragment) OS=Mus musculus GN=Col3a1 PE=2 SV=1 - [Q8K173_MOUSE]	1.72	2	2	2	**2.660**	**3.384**	**2.419**
P11087	Collagen alpha-1(I) chain OS=Mus musculus GN=Col1a1 PE=1 SV=4 - [CO1A1_MOUSE]	3.37	4	4	4	**2.679**	**3.763**	**2.071**
Q04857	Collagen alpha-1(VI) chain OS=Mus musculus GN=Col6a1 PE=1 SV=1 - [CO6A1_MOUSE]	4.59	4	4	5	1.592	**2.931**	1.989
Q61245	Collagen alpha-1(XI) chain OS=Mus musculus GN=Col11a1 PE=1 SV=2 - [COBA1_MOUSE]	1.88	3	4	4	0.782	**2.713**	1.494
P01027	Complement C3 OS=Mus musculus GN=C3 PE=1 SV=3 - [CO3_MOUSE]	7.04	11	11	31	0.793	**5.206**	1.879
B2RWX2	Complement component 4B (Childo blood group) OS=Mus musculus GN=C4b PE=2 SV=1 - [B2RWX2_MOUSE]	1.73	3	3	3	0.556	**2.797**	1.219
H3BJW0	Connective tissue growth factor OS=Mus musculus GN=Ctgf PE=1 SV=1 - [H3BJW0_MOUSE]	7.85	2	2	2	**2.068**	**3.607**	1.892
P12960	Contactin-1 OS=Mus musculus GN=Cntn1 PE=1 SV=1 - [CNTN1_MOUSE]	2.35	2	2	2	1.220	**2.589**	1.602

P62897	Cytochrome c, somatic OS=Mus musculus GN=Cycs PE=1 SV=2 - [CYC_MOUSE]	24.76	3	3	5	1.224	**2.206**	1.538
E9PZW0	Desmoplakin OS=Mus musculus GN=Dsp PE=1 SV=1 - [E9PZW0_MOUSE]	1.27	3	3	3	**0.342**	**2.726**	1.000
Q9QUN9	Dickkopf-related protein 3 OS=Mus musculus GN=Dkk3 PE=2 SV=1 - [DKK3_MOUSE]	16.33	4	4	4	**4.486**	**9.849**	**5.371**
Q8BPB5	EGF-containing fibulin-like extracellular matrix protein 1 OS=Mus musculus GN=Efemp1 PE=1 SV=1 - [FBLN3_MOUSI	4.67	2	2	3	0.888	2.277	0.830
Q9ERV6	Epidermal growth factor receptor isoform 2 OS=Mus musculus GN=Egfr PE=4 SV=1 - [Q9ERV6_MOUSE]	6.69	3	3	3	0.881	2.083	1.047
O09164	Extracellular superoxide dismutase [Cu-Zn] OS=Mus musculus GN=Sod3 PE=1 SV=1 - [SODE_MOUSE]	12.35	2	2	2	**2.340**	**6.509**	**5.747**
B7ZNJ1	Fibronectin OS=Mus musculus GN=Fn1 PE=1 SV=1 - [B7ZNJ1_MOUSE]	3.35	6	6	6	0.850	**2.656**	1.310
Q08879	Fibulin-1 OS=Mus musculus GN=Fbln1 PE=1 SV=2 - [FBLN1_MOUSE]	3.12	2	2	2	0.642	1.132	0.874
Q62356	Follistatin-related protein 1 OS=Mus musculus GN=Fstl1 PE=1 SV=2 - [FSTL1_MOUSE]	5.56	2	2	2	**21.167**	**19.100**	**9.835**
B2RXT5	Glucose-6-phosphate isomerase OS=Mus musculus GN=Gpi1 PE=2 SV=1 - [B2RXT5_MOUSE]	8.43	2	2	3	0.779	**3.186**	1.080
Q61646	Haptoglobin OS=Mus musculus GN=Hp PE=1 SV=1 - [HPT_MOUSE]	11.53	4	4	14	1.499	**3.834**	1.828
P01942	Hemoglobin subunit alpha OS=Mus musculus GN=Hba PE=1 SV=2 - [HBA_MOUSE]	19.01	2	2	5	**0.483**	1.159	1.205
P01868	Ig gamma-1 chain C region secreted form OS=Mus musculus GN=Ighg1 PE=1 SV=1 - [IGHG1_MOUSE]	5.86	2	2	2	0.797	**2.166**	1.757
B7ZWC4	Insulin-like growth factor 2 receptor OS=Mus musculus GN=Igf2r PE=2 SV=1 - [B7ZWC4_MOUSE]	1.13	2	2	2	0.648	**4.921**	1.259
H3BLB7	Insulin-like growth factor-binding protein 4 OS=Mus musculus GN=Igfbp4 PE=1 SV=1 - [H3BLB7_MOUSE]	15.53	2	2	2	**2.102**	**12.270**	**3.305**
P09055	Integrin beta-1 OS=Mus musculus GN=Itgb1 PE=1 SV=1 - [ITB1_MOUSE]	2.38	2	2	2	0.551	**2.296**	1.278
Q61703	Inter-alpha-trypsin inhibitor heavy chain H2 OS=Mus musculus GN=Itih2 PE=1 SV=1 - [ITIH2_MOUSE]	2.01	2	2	2	0.708	**2.153**	1.266
G3UY13	Interleukin-1 receptor accessory protein OS=Mus musculus GN=Il1rap PE=4 SV=1 - [G3UY13_MOUSE]	9.25	3	3	6	1.517	**3.512**	1.617
Q53VP8	Kappa chain (Fragment) OS=Mus musculus PE=2 SV=1 - [Q53VP8_MOUSE]	19.64	2	2	2	0.747	1.516	1.113
B8YJF9	Lactoferrin OS=Mus musculus GN=Ltf PE=2 SV=1 - [B8YJF9_MOUSE]	3.82	3	4	9	**0.453**	**2.087**	1.027
A0A0R4J0I9	Low density lipoprotein receptor-related protein 1 OS=Mus musculus GN=Lrp1 PE=1 SV=1 - [A0A0R4J0I9_MOUSE]	0.46	2	2	2	0.841	1.977	1.216
P51885	Lumican OS=Mus musculus GN=Lum PE=1 SV=2 - [LUM_MOUSE]	11.54	3	3	7	0.851	**2.714**	1.626
B5THE2	Maltase-glucoamylase OS=Mus musculus GN=Mgam PE=1 SV=1 - [B5THE2_MOUSE]	1.31	2	3	7	0.687	1.852	1.226
O70423	Membrane primary amine oxidase OS=Mus musculus GN=Aoc3 PE=1 SV=3 - [AOC3_MOUSE]	5.36	3	3	8	0.641	**2.041**	1.157
P02798	Metallothionein-2 OS=Mus musculus GN=Mt2 PE=1 SV=2 - [MT2_MOUSE]	21.31	2	2	3	0.530	0.740	0.746
E9Q589	Neural cell adhesion molecule 1 OS=Mus musculus GN=Ncam1 PE=1 SV=1 - [E9Q589_MOUSE]	3.31	2	2	5	0.804	**2.945**	1.251
Q02819	Nucleobindin-1 OS=Mus musculus GN=Nucb1 PE=1 SV=2 - [NUCB1_MOUSE]	23.97	9	9	11	**4.225**	**17.923**	**5.085**
P20918	Plasminogen OS=Mus musculus GN=Plg PE=1 SV=3 - [PLMN_MOUSE]	4.56	2	2	3	**0.476**	1.498	0.846
E0CXB1	Proteasome subunit alpha type-6 OS=Mus musculus GN=Psma6 PE=1 SV=1 - [E0CXB1_MOUSE]	10.13	2	2	2	1.512	**2.245**	**2.740**
Q3UDR2	Protein disulfide-isomerase (Fragment) OS=Mus musculus GN=P4hb PE=2 SV=1 - [Q3UDR2_MOUSE]	9.11	4	4	4	0.901	2.890	1.531
B8JJN0	Protein Gm20547 OS=Mus musculus GN=Gm20547 PE=4 SV=1 - [B8JJN0_MOUSE]	3.37	3	3	5	0.603	**2.969**	1.528
A0A0J9YVE6	Protein Mgam OS=Mus musculus GN=Mgam PE=1 SV=1 - [A0A0J9YVE6_MOUSE]	2.42	2	3	6	0.758	1.945	1.216
H7BX99	Prothrombin OS=Mus musculus GN=F2 PE=1 SV=1 - [H7BX99_MOUSE]	5.51	2	2	7	0.841	**2.195**	1.197
Q3UD81	Putative uncharacterized protein (Fragment) OS=Mus musculus GN=Actg1 PE=2 SV=1 - [Q3UD81_MOUSE]	13.19	3	3	6	0.985	**4.595**	1.301
Q3UWP8	Putative uncharacterized protein (Fragment) OS=Mus musculus GN=Calr PE=2 SV=1 - [Q3UWP8_MOUSE]	18.75	5	5	5	1.135	**5.427**	**2.098**
Q3TP88	Putative uncharacterized protein (Fragment) OS=Mus musculus GN=Col1a2 PE=2 SV=1 - [Q3TP88_MOUSE]	2.71	2	2	2	**5.488**	**6.549**	**3.497**
Q3TR43	Putative uncharacterized protein OS=Mus musculus GN=Dpp4 PE=2 SV=1 - [Q3TR43_MOUSE]	2.33	2	2	3	0.629	1.954	1.417
Q3TXQ4	Putative uncharacterized protein OS=Mus musculus GN=Npc2 PE=2 SV=1 - [Q3TXQ4_MOUSE]	16.78	2	2	2	**39.939**	**60.295**	**44.861**

F8VQD7	Receptor-type tyrosine-protein phosphatase gamma OS=Mus musculus GN=Ptprg PE=1 SV=1 - [F8VQD7_MOUSE]	2.50	3	3	3	0.820	**2.189**	1.415
Q64374	Regucalcin OS=Mus musculus GN=Rgn PE=1 SV=1 - [RGN_MOUSE]	12.37	3	3	3	0.604	**2.322**	1.874
Q9QUR8	Semaphorin-7A OS=Mus musculus GN=Sema7a PE=1 SV=1 - [SEM7A_MOUSE]	3.16	2	2	2	0.895	2.553	1.144
Q921I1	Serotransferrin OS=Mus musculus GN=Tf PE=1 SV=1 - [TRFE_MOUSE]	21.23	16	17	64	0.704	**2.488**	1.165
P07724	Serum albumin OS=Mus musculus GN=Alb PE=1 SV=3 - [ALBU_MOUSE]	4.44	2	2	25	0.631	**3.391**	1.486
Q91VW3	SH3 domain-binding glutamic acid-rich-like protein 3 OS=Mus musculus GN=Sh3bgrl3 PE=1 SV=1 - [SH3L3_MOUSE]	26.88	2	2	4	0.879	1.394	1.054
Q5NCU4	SPARC OS=Mus musculus GN=Sparc PE=1 SV=1 - [Q5NCU4_MOUSE]	43.85	11	11	20	**6.504**	**12.783**	**7.595**
P08228	Superoxide dismutase [Cu-Zn] OS=Mus musculus GN=Sod1 PE=1 SV=2 - [SODC_MOUSE]	24.68	4	4	5	1.681	**2.434**	1.514
G4XXT0	Testis-specific cytochrome c (Fragment) OS=Mus musculus GN=CYCt PE=3 SV=1 - [G4XXT0_MOUSE]	26.79	2	2	3	0.804	1.693	0.976
P17751	Triosephosphate isomerase OS=Mus musculus GN=Tpi1 PE=1 SV=4 - [TPIS_MOUSE]	16.72	4	4	8	0.622	1.512	0.936
A2AIM4	Tropomyosin beta chain OS=Mus musculus GN=Tpm2 PE=1 SV=1 - [A2AIM4_MOUSE]	7.39	2	2	2	1.796	**3.617**	1.443
P35917	Vascular endothelial growth factor receptor 3 OS=Mus musculus GN=Flt4 PE=1 SV=1 - [VGFR3_MOUSE]	1.69	2	2	2	0.756	**3.102**	1.240
A0A0A6YWC8	Vimentin OS=Mus musculus GN=Vim PE=1 SV=1 - [A0A0A6YWC8_MOUSE]	7.96	3	3	3	**4.065**	**6.724**	**2.217**
O08532	Voltage-dependent calcium channel subunit alpha-2/delta-1 OS=Mus musculus GN=Cacna2d1 PE=1 SV=1 - [CA2D1_	11.79	11	11	12	0.728	**2.224**	1.130
Q11136	Xaa-Pro dipeptidase OS=Mus musculus GN=Pepd PE=1 SV=3 - [PEPD_MOUSE]	16.63	7	7	8	0.896	**2.859**	1.373

Supplementary Table 3B

Accession	Description	Coverage	Unique Peptides	Peptides	PSMs	P OP9/Ctrl	S OP9/Ctrl	T OP9/Ctrl
P62258	14-3-3 protein epsilon OS=Homo sapiens GN=YWHAE PE=1 SV=1 - [1433E_HUMAN]	22.35	3	6	7	1.516	**2.865**	**2.141**
P63104	14-3-3 protein zeta/delta OS=Homo sapiens GN=YWHAZ PE=1 SV=1 - [1433Z_HUMAN]	18.37	2	5	6	1.162	**3.505**	1.028
I3L3I0	Actin, cytoplasmic 2 (Fragment) OS=Homo sapiens GN=ACTG1 PE=1 SV=1 - [I3L3I0_HUMAN]	14.49	3	3	6	0.985	**4.595**	1.301
H0Y984	ADP-ribosyl cyclase/cyclic ADP-ribose hydrolase 2 (Fragment) OS=Homo sapiens GN=BST1 PE=1 SV=1 - [H0Y984_HUMAN]	11.83	2	2	2	0.773	1.951	1.321
P43652	Afamin OS=Homo sapiens GN=AFM PE=1 SV=1 - [AFAM_HUMAN]	39.57	23	23	31	0.679	**2.686**	1.273
Q6PID9	Aggrecan OS=Homo sapiens GN=ACAN PE=1 SV=1 - [Q6PID9_HUMAN]	4.30	3	3	3	0.883	1.758	1.036
P02763	Alpha-1-acid glycoprotein 1 OS=Homo sapiens GN=ORM1 PE=1 SV=1 - [A1AG1_HUMAN]	45.27	7	10	28	0.694	1.381	1.535
P19652	Alpha-1-acid glycoprotein 2 OS=Homo sapiens GN=ORM2 PE=1 SV=2 - [A1AG2_HUMAN]	43.78	6	9	15	0.591	1.310	1.250
P01011	Alpha-1-antichymotrypsin OS=Homo sapiens GN=SERPINA3 PE=1 SV=2 - [AACT_HUMAN]	25.06	10	10	16	0.676	**3.225**	1.400
P01009	Alpha-1-antitrypsin OS=Homo sapiens GN=SERPINA1 PE=1 SV=3 - [A1AT_HUMAN]	50.00	2	23	57	**0.426**	1.505	**2.540**
P04217	Alpha-1B-glycoprotein OS=Homo sapiens GN=A1BG PE=1 SV=4 - [A1BG_HUMAN]	34.95	12	12	40	0.756	**2.407**	1.313
P08697	Alpha-2-antiplasmin OS=Homo sapiens GN=SERPINF2 PE=1 SV=3 - [A2AP_HUMAN]	22.00	11	11	14	0.813	**2.385**	1.359
P02765	Alpha-2-HS-glycoprotein OS=Homo sapiens GN=AHSG PE=1 SV=1 - [FETUA_HUMAN]	55.31	18	18	68	0.928	**3.507**	1.433
P01023	Alpha-2-macroglobulin OS=Homo sapiens GN=A2M PE=1 SV=3 - [A2MG_HUMAN]	40.50	39	42	75	**0.477**	1.331	**2.089**
Q6NSB3	Alpha-amylase (Fragment) OS=Homo sapiens GN=AMY1A PE=2 SV=1 - [Q6NSB3_HUMAN]	13.74	6	6	7	1.519	**3.621**	**2.324**
P15144	Aminopeptidase N OS=Homo sapiens GN=ANPEP PE=1 SV=4 - [AMPN_HUMAN]	10.86	11	11	11	0.716	**2.346**	1.255
P01008	Antithrombin-III OS=Homo sapiens GN=SERPINC1 PE=1 SV=1 - [ANT3_HUMAN]	49.78	23	23	77	0.634	1.845	1.076
P02647	Apolipoprotein A-I OS=Homo sapiens GN=APOA1 PE=1 SV=1 - [APOA1_HUMAN]	39.33	10	10	15	**0.321**	1.002	0.685
V9GYE3	Apolipoprotein A-II OS=Homo sapiens GN=APOA2 PE=1 SV=1 - [V9GYE3_HUMAN]	40.38	3	3	3	**0.460**	1.304	0.783
P06727	Apolipoprotein A-IV OS=Homo sapiens GN=APOA4 PE=1 SV=3 - [APOA4_HUMAN]	75.00	33	33	87	0.836	**3.060**	1.450
A0A0S2Z3V0	Apolipoprotein E isoform 2 (Fragment) OS=Homo sapiens GN=APOE PE=2 SV=1 - [A0A0S2Z3V0_HUMAN]	8.47	2	2	3	0.910	**2.436**	1.063
Q13790	Apolipoprotein F OS=Homo sapiens GN=APOF PE=1 SV=2 - [APOF_HUMAN]	8.28	2	2	2	0.914	**2.403**	1.376
P08519	Apolipoprotein(a) OS=Homo sapiens GN=LPA PE=1 SV=1 - [APOA_HUMAN]	7.30	2	2	5	1.000	**2.887**	1.232
B7Z1I2	Aspartate aminotransferase OS=Homo sapiens PE=2 SV=1 - [B7Z1I2_HUMAN]	5.74	2	2	2	0.808	**3.594**	1.409
P25705	ATP synthase subunit alpha, mitochondrial OS=Homo sapiens GN=ATP5A1 PE=1 SV=1 - [ATPA_HUMAN]	3.62	2	2	2	**0.440**	1.903	1.145
O75882	Attractin OS=Homo sapiens GN=ATRN PE=1 SV=2 - [ATRN_HUMAN]	24.42	31	31	51	0.758	**2.148**	1.235
H0YLF3	Beta-2-microglobulin (Fragment) OS=Homo sapiens GN=B2M PE=1 SV=1 - [H0YLF3_HUMAN]	28.17	2	2	2	0.825	**3.829**	1.121
P43251	Biotinidase OS=Homo sapiens GN=BTD PE=1 SV=2 - [BTD_HUMAN]	20.44	9	9	20	0.724	**2.156**	1.177
K7ESE8	Bleomycin hydrolase (Fragment) OS=Homo sapiens GN=BLMH PE=1 SV=1 - [K7ESE8_HUMAN]	12.55	3	3	3	0.627	**2.156**	1.410
A0A087X0X6	Cadherin-13 OS=Homo sapiens GN=CDH13 PE=1 SV=1 - [A0A087X0X6_HUMAN]	12.86	6	6	6	1.125	**2.007**	0.954
Q9BRL5	CALM3 protein OS=Homo sapiens PE=1 SV=1 - [Q9BRL5_HUMAN]	19.05	3	3	3	1.714	**2.567**	1.761
Q53G71	Calreticulin variant (Fragment) OS=Homo sapiens PE=2 SV=1 - [Q53G71_HUMAN]	21.18	8	8	9	1.133	**4.206**	**2.098**
A0A024CIM4	Carboxylic ester hydrolase OS=Homo sapiens PE=3 SV=1 - [A0A024CIM4_HUMAN]	22.92	14	14	18	0.732	**2.290**	1.236
Q9Y646	Carboxypeptidase Q OS=Homo sapiens GN=CPQ PE=1 SV=1 - [CBPQ_HUMAN]	5.72	2	2	2	0.728	**2.250**	1.307
Q5T4F6	Cartilage acidic protein 1 (Fragment) OS=Homo sapiens GN=CRTAC1 PE=1 SV=1 - [Q5T4F6_HUMAN]	5.92	2	2	2	0.955	**2.217**	1.620
P31944	Caspase-14 OS=Homo sapiens GN=CASP14 PE=1 SV=2 - [CASPE_HUMAN]	14.05	3	3	3	1.224	1.589	**2.878**
Q5U000	Cathepsin Z OS=Homo sapiens PE=2 SV=1 - [Q5U000_HUMAN]	7.26	2	2	2	0.524	**3.164**	1.818

Q6YHK3	CD109 antigen OS=Homo sapiens GN=CD109 PE=1 SV=2 - [CD109_HUMAN]	1.11	2	2	2	0.899	1.976	1.330
F5GXJ9	CD166 antigen OS=Homo sapiens GN=ALCAM PE=1 SV=1 - [F5GXJ9_HUMAN]	13.53	6	6	6	0.794	1.861	1.103
H0YD13	CD44 antigen OS=Homo sapiens GN=CD44 PE=1 SV=2 - [H0YD13_HUMAN]	14.08	3	3	4	0.830	1.656	1.085
B3KNB4	cDNA FLJ14168 fis, clone NT2RP2001440, highly similar to 14-3-3 protein gamma OS=Homo sapiens PE=2 SV=1 - [B3KNB4_HUMAN]	26.72	4	7	8	**3.814**	**5.661**	**5.400**
Q8N294	cDNA FLJ33633 fis, clone BRAMY2022786, highly similar to Homo sapiens dickkopf-3 (DKK-3) mRNA OS=Homo sapiens PE=2 SV=1 - [Q8N294_HUMAN]	16.28	2	2	2	**4.412**	**8.912**	**3.526**
B4DPR2	cDNA FLJ50830, highly similar to Serum albumin OS=Homo sapiens PE=2 SV=1 - [B4DPR2_HUMAN]	24.09	16	16	123	0.631	**3.250**	1.223
B4DPN0	cDNA FLJ51265, moderately similar to Beta-2-glycoprotein 1 (Beta-2-glycoprotein I) OS=Homo sapiens PE=2 SV=1 - [B4DPN0_HUMAN]	19.34	4	4	4	0.540	**3.972**	0.933
B4DPP8	cDNA FLJ53075, highly similar to Kininogen-1 OS=Homo sapiens PE=2 SV=1 - [B4DPP8_HUMAN]	46.75	2	26	61	1.501	**4.368**	1.356
B4DNT6	cDNA FLJ53631, highly similar to Intercellular adhesion molecule 1 OS=Homo sapiens PE=2 SV=1 - [B4DNT6_HUMAN]	7.01	3	3	3	0.886	1.706	1.181
B4E1B2	cDNA FLJ53691, highly similar to Serotransferrin OS=Homo sapiens PE=2 SV=1 - [B4E1B2_HUMAN]	86.14	2	78	1208	0.661	**2.452**	1.089
B4E1B3	cDNA FLJ53950, highly similar to Angiotensinogen OS=Homo sapiens PE=2 SV=1 - [B4E1B3_HUMAN]	26.61	10	10	31	0.838	**2.574**	1.421
B4DVY2	cDNA FLJ54184, highly similar to Tropomyosin alpha-4 chain OS=Homo sapiens PE=2 SV=1 - [B4DVY2_HUMAN]	12.66	3	3	3	1.653	**3.688**	1.896
B4E1I8	cDNA FLJ54228, highly similar to Leucine-rich alpha-2-glycoprotein OS=Homo sapiens PE=2 SV=1 - [B4E1I8_HUMAN]	26.97	7	7	7	0.900	**2.994**	1.346
B4DNS6	cDNA FLJ54278, highly similar to SPARC-like protein 1 OS=Homo sapiens PE=2 SV=1 - [B4DNS6_HUMAN]	7.51	3	3	3	0.740	**2.224**	1.049
B4DUI7	cDNA FLJ55004, highly similar to Complement-activating component of Ra-reactive factor (EC 3.4.21.-) OS=Homo sapiens PE=2 SV=1 - [B4DUI7_HUMAI	5.93	2	2	3	0.875	**2.621**	1.590
B4DSV9	cDNA FLJ56632, moderately similar to Target of Nesh-SH3 OS=Homo sapiens PE=2 SV=1 - [B4DSV9_HUMAN]	4.18	3	3	3	1.124	**3.762**	1.434
B4DMX4	cDNA FLJ57154, highly similar to Alpha-fetoprotein OS=Homo sapiens PE=2 SV=1 - [B4DMX4_HUMAN]	10.42	6	6	13	**0.207**	0.638	**0.415**
B4DNH0	cDNA FLJ60313, highly similar to Epithelial-cadherin OS=Homo sapiens PE=2 SV=1 - [B4DNH0_HUMAN]	4.84	2	2	2	0.897	1.409	1.067
A8K335	cDNA FLJ76254, highly similar to Homo sapiens gamma-glutamyl hydrolase (GGH), mRNA OS=Homo sapiens PE=2 SV=1 - [A8K335_HUMAN]	11.01	3	3	4	0.832	**2.548**	1.453
A8K5A4	cDNA FLJ76826, highly similar to Homo sapiens ceruloplasmin (ferroxidase) (CP), mRNA OS=Homo sapiens PE=2 SV=1 - [A8K5A4_HUMAN]	51.17	4	49	214	0.652	**2.034**	1.176
A8K6K4	cDNA FLJ77565, highly similar to Homo sapiens IL1RAP, transcript variant 1, mRNA OS=Homo sapiens PE=2 SV=1 - [A8K6K4_HUMAN]	8.42	6	6	11	1.309	**3.191**	1.456
A8K9A9	cDNA FLJ77744, highly similar to Homo sapiens kallikrein B, plasma (Fletcher factor) 1 (KLKB1), mRNA OS=Homo sapiens PE=2 SV=1 - [A8K9A9_HUMAI	3.92	3	3	3	1.073	**3.328**	1.126
A8K7Q1	cDNA FLJ77770, highly similar to Homo sapiens nucleobindin 1 (NUCB1), mRNA OS=Homo sapiens PE=2 SV=1 - [A8K7Q1_HUMAN]	22.78	9	9	12	**3.791**	**14.617**	**4.548**
A8KAJ3	cDNA FLJ77823, highly similar to Homo sapiens EGF-containing FLECM protein 1, variant 3, mRNA OS=Homo sapiens PE=2 SV=1 - [A8KAJ3_HUMAN]	12.17	5	5	7	0.888	1.213	1.000
B7ZAL5	cDNA, FLJ79229, highly similar to Lactotransferrin (EC 3.4.21.-) OS=Homo sapiens PE=2 SV=1 - [B7ZAL5_HUMAN]	12.01	7	7	107	0.714	1.713	1.124
B2R773	cDNA, FLJ93312, highly similar to Homo sapiens adipose most abundant gene transcript 1 (APM1), mRNA OS=Homo sapiens PE=2 SV=1 - [B2R773_HUN	12.30	2	2	4	1.625	**14.310**	**3.742**
B2R9F2	cDNA, FLJ94361, highly similar to Homo sapiens SERPINA6, mRNA OS=Homo sapiens PE=2 SV=1 - [B2R9F2_HUMAN]	28.64	9	9	15	0.786	**2.456**	1.326
B2RAN2	cDNA, FLJ95014, highly similar to Homo sapiens vanin 1 (VNN1), mRNA OS=Homo sapiens PE=2 SV=1 - [B2RAN2_HUMAN]	17.54	7	7	17	0.748	1.987	1.252
A0A087X0T8	Cell adhesion molecule 1 OS=Homo sapiens GN=CADM1 PE=1 SV=1 - [A0A087X0T8_HUMAN]	26.30	6	6	7	0.827	1.746	1.023
G5E968	Chromogranin A (Parathyroid secretory protein 1), isoform CRA_b OS=Homo sapiens GN=CHGA PE=1 SV=1 - [G5E968_HUMAN]	9.15	2	2	2	1.009	1.342	0.915
P10909	Clusterin OS=Homo sapiens GN=CLU PE=1 SV=1 - [CLUS_HUMAN]	9.35	4	4	4	0.701	**2.210**	1.163
Q14019	Coactosin-like protein OS=Homo sapiens GN=COTL1 PE=1 SV=3 - [COTL1_HUMAN]	22.54	4	4	4	0.822	**2.723**	1.449
P12259	Coagulation factor V OS=Homo sapiens GN=F5 PE=1 SV=4 - [FA5_HUMAN]	1.03	2	2	3	0.976	**2.199**	0.968
P00742	Coagulation factor X OS=Homo sapiens GN=F10 PE=1 SV=2 - [FA10_HUMAN]	11.48	5	5	7	1.791	**3.294**	1.840
P02461	Collagen alpha-1(III) chain OS=Homo sapiens GN=COL3A1 PE=1 SV=4 - [CO3A1_HUMAN]	2.18	3	3	5	0.824	**2.454**	1.356
A0A087X0S5	Collagen alpha-1(VI) chain OS=Homo sapiens GN=COL6A1 PE=1 SV=1 - [A0A087X0S5_HUMAN]	2.44	2	2	2	1.366	**3.747**	**2.916**
D3DTX7	Collagen, type I, alpha 1, isoform CRA_a OS=Homo sapiens GN=COL1A1 PE=4 SV=1 - [D3DTX7_HUMAN]	4.63	4	4	4	**2.244**	1.515	1.183
D3DT71	Collagen, type XI, alpha 1, isoform CRA_b OS=Homo sapiens GN=COL11A1 PE=4 SV=1 - [D3DT71_HUMAN]	2.32	4	5	5	0.674	1.418	1.156

B4DQI1	Complement C2 OS=Homo sapiens GN=C2 PE=1 SV=1 - [B4DQI1_HUMAN]	40.51	5	20	43	0.725	**2.596**	1.352
P01024	Complement C3 OS=Homo sapiens GN=C3 PE=1 SV=2 - [CO3_HUMAN]	53.22	79	79	236	0.822	**6.256**	**2.086**
A0A0G2JPR0	Complement C4-A OS=Homo sapiens GN=C4A PE=1 SV=1 - [A0A0G2JPR0_HUMAN]	9.29	2	12	17	0.710	**4.138**	1.168
G3XAM2	Complement factor I OS=Homo sapiens GN=CFI PE=1 SV=1 - [G3XAM2_HUMAN]	22.05	3	10	12	**0.410**	**2.389**	1.379
Q12860	Contactin-1 OS=Homo sapiens GN=CNTN1 PE=1 SV=1 - [CNTN1_HUMAN]	2.36	2	2	2	1.220	**2.589**	1.602
P01040	Cystatin-A OS=Homo sapiens GN=CSTA PE=1 SV=1 - [CYTA_HUMAN]	25.51	2	2	2	0.929	1.130	1.177
P54108	Cysteine-rich secretory protein 3 OS=Homo sapiens GN=CRISP3 PE=1 SV=1 - [CRIS3_HUMAN]	16.73	4	4	6	0.772	1.639	1.194
C9JFR7	Cytochrome c (Fragment) OS=Homo sapiens GN=CYCS PE=1 SV=1 - [C9JFR7_HUMAN]	18.81	2	2	4	1.110	**2.109**	1.389
P81605	Dermcidin OS=Homo sapiens GN=DCD PE=1 SV=2 - [DCD_HUMAN]	20.00	3	3	4	**0.440**	0.780	1.388
Q14117	Dihydropyrimidinase OS=Homo sapiens GN=DPYS PE=1 SV=1 - [DPYS_HUMAN]	3.08	2	2	2	0.861	**2.633**	1.405
H0YCY8	Dipeptidyl peptidase 1 (Fragment) OS=Homo sapiens GN=CTSC PE=1 SV=7 - [H0YCY8_HUMAN]	7.35	2	2	3	0.544	1.687	0.825
P27487	Dipeptidyl peptidase 4 OS=Homo sapiens GN=DPP4 PE=1 SV=2 - [DPP4_HUMAN]	8.62	7	7	10	0.611	**2.035**	1.210
P09172	Dopamine beta-hydroxylase OS=Homo sapiens GN=DBH PE=1 SV=3 - [DOPO_HUMAN]	8.27	4	4	4	0.675	**2.043**	1.150
Q4LE79	DSP variant protein (Fragment) OS=Homo sapiens GN=DSP variant protein PE=2 SV=1 - [Q4LE79_HUMAN]	2.76	7	7	7	**0.330**	1.109	1.025
Q68GS6	Epidermal growth factor receptor (Fragment) OS=Homo sapiens GN=EGFR PE=2 SV=1 - [Q68GS6_HUMAN]	5.43	3	3	3	0.564	**2.083**	1.307
Q8WWE4	FGL2 protein OS=Homo sapiens PE=2 SV=1 - [Q8WWE4_HUMAN]	12.84	3	3	3	1.237	**2.289**	1.372
P23142	Fibulin-1 OS=Homo sapiens GN=FBLN1 PE=1 SV=4 - [FBLN1_HUMAN]	6.97	5	5	5	0.745	1.182	0.827
B7ZLE5	FN1 protein OS=Homo sapiens GN=FN1 PE=2 SV=1 - [B7ZLE5_HUMAN]	4.51	9	9	9	0.842	**2.036**	1.187
Q12841	Follistatin-related protein 1 OS=Homo sapiens GN=FSTL1 PE=1 SV=1 - [FSTL1_HUMAN]	17.21	6	6	7	**3.321**	**6.862**	**2.824**
B7UCU6	Gamma-globin chain (Fragment) OS=Homo sapiens GN=HBG2 PE=3 SV=1 - [B7UCU6_HUMAN]	23.81	2	3	7	0.612	1.341	0.869
P06396	Gelsolin OS=Homo sapiens GN=GSN PE=1 SV=1 - [GELS_HUMAN]	3.71	3	3	5	0.582	1.733	1.166
B4DE36	Glucose-6-phosphate isomerase OS=Homo sapiens PE=2 SV=1 - [B4DE36_HUMAN]	6.98	3	3	4	0.785	**2.706**	1.243
B0BCY7	Glutamyl aminopeptidase (Aminopeptidase A) OS=Homo sapiens GN=ENPEP PE=2 SV=1 - [B0BCY7_HUMAN]	1.78	2	2	2	0.947	**3.467**	1.494
A0A087X1J7	Glutathione peroxidase OS=Homo sapiens GN=GPX3 PE=1 SV=1 - [A0A087X1J7_HUMAN]	35.56	7	7	8	**0.348**	0.801	**0.465**
Q8NBJ4	Golgi membrane protein 1 OS=Homo sapiens GN=GOLM1 PE=1 SV=1 - [GOLM1_HUMAN]	4.74	2	2	3	0.582	1.985	1.372
P00738	Haptoglobin OS=Homo sapiens GN=HP PE=1 SV=1 - [HPT_HUMAN]	58.62	25	25	62	**0.490**	1.237	**2.102**
P68871	Hemoglobin subunit beta OS=Homo sapiens GN=HBB PE=1 SV=2 - [HBB_HUMAN]	81.63	7	9	13	**0.430**	0.947	1.820
A0A024RAB6	Heparan sulfate proteoglycan 2 (Perlecan), isoform CRA_b OS=Homo sapiens GN=HSPG2 PE=4 SV=1 - [A0A024RAB6_HUMAN]	0.41	2	2	2	0.972	**3.106**	1.480
Q04756	Hepatocyte growth factor activator OS=Homo sapiens GN=HGFAC PE=1 SV=1 - [HGFA_HUMAN]	4.27	3	3	3	0.791	1.901	1.278
Q86YZ3	Hornerin OS=Homo sapiens GN=HRNR PE=1 SV=2 - [HORN_HUMAN]	3.05	4	4	4	1.000	1.000	0.950
P01861	Ig gamma-4 chain C region OS=Homo sapiens GN=IGHG4 PE=1 SV=1 - [IGHG4_HUMAN]	37.00	3	9	15	0.522	1.423	1.466
P01608	Ig kappa chain V-I region Roy OS=Homo sapiens PE=1 SV=1 - [KV116_HUMAN]	24.07	2	2	2	**0.401**	1.218	**2.058**
P01621	Ig kappa chain V-III region NG9 (Fragment) OS=Homo sapiens PE=2 SV=1 - [KV303_HUMAN]	25.00	2	2	2	0.714	1.395	1.076
Q9Y6R7	IgGFc-binding protein OS=Homo sapiens GN=FCGBP PE=1 SV=3 - [FCGBP_HUMAN]	0.65	2	2	2	0.706	1.644	0.740
Q6PJF2	IGK@ protein OS=Homo sapiens GN=IGK@ PE=1 SV=1 - [Q6PJF2_HUMAN]	42.98	3	6	16	**0.372**	1.157	**2.885**
Q8N355	IGL@ protein OS=Homo sapiens GN=IGL@ PE=1 SV=1 - [Q8N355_HUMAN]	46.58	3	10	21	0.620	1.440	0.910
Q9NPP6	Immunoglobulin heavy chain variant (Fragment) OS=Homo sapiens PE=2 SV=1 - [Q9NPP6_HUMAN]	29.33	3	8	12	**0.352**	1.232	**2.830**
F6MZK5	Insulin (Fragment) OS=Homo sapiens GN=INS PE=2 SV=1 - [F6MZK5_HUMAN]	29.79	2	2	4	0.878	1.606	1.355

Q59EZ3	Insulin-like growth factor 2 receptor variant (Fragment) OS=Homo sapiens PE=1 SV=1 - [Q59EZ3_HUMAN]	1.33	4	4	5	0.615	**2.560**	1.352
P05556	Integrin beta-1 OS=Homo sapiens GN=ITGB1 PE=1 SV=2 - [ITB1_HUMAN]	2.38	2	2	3	0.704	**2.609**	1.363
B2RMS9	Inter-alpha (Globulin) inhibitor H4 (Plasma Kallikrein-sensitive glycoprotein) OS=Homo sapiens GN=ITIH4 PE=2 SV=1 - [B2RMS9_HUMAN]	16.99	14	14	21	0.799	**2.741**	1.526
J3QQR8	Intercellular adhesion molecule 2 (Fragment) OS=Homo sapiens GN=ICAM2 PE=1 SV=7 - [J3QQR8_HUMAN]	14.97	2	2	4	0.704	1.876	1.146
P08637	Low affinity immunoglobulin gamma Fc region receptor III-A OS=Homo sapiens GN=FCGR3A PE=1 SV=2 - [FCG3A_HUMAN]	12.20	4	4	4	0.892	**2.051**	1.172
P51884	Lumican OS=Homo sapiens GN=LUM PE=1 SV=2 - [LUM_HUMAN]	17.46	4	4	9	0.890	**2.778**	1.626
Q8TE24	Maltase-glucoamylase (Fragment) OS=Homo sapiens GN=MGAM PE=3 SV=1 - [Q8TE24_HUMAN]	5.19	8	11	28	0.738	**2.011**	1.266
Q16853	Membrane primary amine oxidase OS=Homo sapiens GN=AOC3 PE=1 SV=3 - [AOC3_HUMAN]	5.24	3	3	7	0.657	1.975	1.177
A0A0K2BMD8	Mutant hemoglobin alpha 2 globin chain OS=Homo sapiens GN=HBA2 PE=3 SV=1 - [A0A0K2BMD8_HUMAN]	57.04	5	5	9	**0.495**	1.588	0.967
P02689	Myelin P2 protein OS=Homo sapiens GN=PMP2 PE=1 SV=3 - [MYP2_HUMAN]	14.39	2	2	2	**8.713**	**24.442**	**9.778**
P13535	Myosin-8 OS=Homo sapiens GN=MYH8 PE=1 SV=3 - [MYH8_HUMAN]	0.88	2	2	2	0.714	**2.842**	1.121
Q96PD5	N-acetylmuramoyl-L-alanine amidase OS=Homo sapiens GN=PGLYRP2 PE=1 SV=1 - [PGRP2_HUMAN]	6.08	2	2	2	0.669	**2.658**	1.480
A0A087X0M8	Neural cell adhesion molecule L1-like protein OS=Homo sapiens GN=CHL1 PE=1 SV=1 - [A0A087X0M8_HUMAN]	2.48	2	2	2	0.908	**2.479**	1.218
J3K000	PEPD protein OS=Homo sapiens GN=PEPD PE=2 SV=1 - [J3K000_HUMAN]	10.34	4	4	4	0.674	**2.844**	1.287
Q6UXB8	Peptidase inhibitor 16 OS=Homo sapiens GN=PI16 PE=1 SV=1 - [PI16_HUMAN]	8.21	4	4	5	0.870	**2.054**	1.201
A0A0F7G8J1	Plasminogen OS=Homo sapiens GN=PLG PE=2 SV=1 - [A0A0F7G8J1_HUMAN]	7.42	5	6	11	0.582	1.471	0.885
P21246	Pleiotrophin OS=Homo sapiens GN=PTN PE=1 SV=1 - [PTN_HUMAN]	10.71	2	2	2	1.315	**12.138**	**4.589**
Q6SYC2	Poliovirus receptor-related 1 (Fragment) OS=Homo sapiens GN=PVRL1 PE=4 SV=1 - [Q6SYC2_HUMAN]	9.13	2	2	2	0.869	**3.661**	1.276
F5H265	Polyubiquitin-C (Fragment) OS=Homo sapiens GN=UBC PE=1 SV=1 - [F5H265_HUMAN]	33.56	2	2	3	0.920	1.498	0.930
Q59EE7	Pro-alpha-1 type V collagen variant (Fragment) OS=Homo sapiens PE=2 SV=1 - [Q59EE7_HUMAN]	2.62	3	4	4	0.721	**2.274**	0.995
Q2M2H8	Probable maltase-glucoamylase 2 OS=Homo sapiens GN=MGAM2 PE=2 SV=3 - [MGAL_HUMAN]	2.19	3	5	15	0.603	**2.112**	1.143
Q5J7V8	Proliferation-inducing gene 20 protein OS=Homo sapiens PE=2 SV=1 - [Q5J7V8_HUMAN]	19.39	2	2	2	**0.192**	**2.359**	1.366
Q07954	Prolow-density lipoprotein receptor-related protein 1 OS=Homo sapiens GN=LRP1 PE=1 SV=2 - [LRP1_HUMAN]	0.46	2	2	2	0.841	1.977	1.216
P41222	Prostaglandin-H2 D-isomerase OS=Homo sapiens GN=PTGDS PE=1 SV=1 - [PTGDS_HUMAN]	12.63	2	2	2	0.760	1.984	0.920
G3V295	Proteasome subunit alpha type OS=Homo sapiens GN=PSMA6 PE=1 SV=1 - [G3V295_HUMAN]	11.33	2	2	2	1.512	**2.245**	**2.740**
P02760	Protein AMBP OS=Homo sapiens GN=AMBP PE=1 SV=1 - [AMBP_HUMAN]	36.36	11	11	49	1.584	**4.837**	**2.040**
C9IZG4	Protein CutA OS=Homo sapiens GN=CUTA PE=1 SV=1 - [C9IZG4_HUMAN]	20.74	2	2	2	0.721	1.622	1.226
Q99497	Protein deglycase DJ-1 OS=Homo sapiens GN=PARK7 PE=1 SV=2 - [PARK7_HUMAN]	18.52	2	2	2	0.582	**2.696**	0.927
B3KQT9	Protein disulfide-isomerase OS=Homo sapiens PE=2 SV=1 - [B3KQT9_HUMAN]	4.38	2	2	2	0.672	**3.464**	1.998
B4DNL5	Protein disulfide-isomerase OS=Homo sapiens PE=2 SV=1 - [B4DNL5_HUMAN]	9.35	4	4	4	0.901	**2.890**	1.531
Q9ULI3	Protein HEG homolog 1 OS=Homo sapiens GN=HEG1 PE=1 SV=3 - [HEG1_HUMAN]	1.16	2	2	3	0.832	1.560	1.292
A0A087WW89	Protein IGHV3-72 OS=Homo sapiens GN=IGHV3-72 PE=1 SV=1 - [A0A087WW89_HUMAN]	29.70	2	2	4	0.519	1.519	1.834
A0A0A0MTQ6	Protein IGKV2D-28 OS=Homo sapiens GN=IGKV2D-28 PE=1 SV=1 - [A0A0A0MTQ6_HUMAN]	36.27	2	2	2	0.553	1.954	1.344
P31151	Protein S100-A7 OS=Homo sapiens GN=S100A7 PE=1 SV=4 - [S10A7_HUMAN]	24.75	2	2	2	1.478	1.096	1.470
A0A096LPE2	Protein SAA2-SAA4 OS=Homo sapiens GN=SAA2-SAA4 PE=4 SV=1 - [A0A096LPE2_HUMAN]	8.17	2	2	2	0.515	1.445	**0.298**
A0A0C4DFV9	Protein SET OS=Homo sapiens GN=SET PE=1 SV=1 - [A0A0C4DFV9_HUMAN]	6.39	2	2	2	**2.181**	**2.910**	1.062
A0A0U1RR20	Proteoglycan 4 OS=Homo sapiens GN=PRG4 PE=1 SV=1 - [A0A0U1RR20_HUMAN]	15.50	10	10	13	0.832	1.815	1.033
E9PIT3	Prothrombin OS=Homo sapiens GN=F2 PE=1 SV=1 - [E9PIT3_HUMAN]	30.87	15	15	61	0.805	**2.052**	1.136

Q5BJH1	PSAP protein OS=Homo sapiens GN=PSAP PE=1 SV=1 - [Q5BJH1_HUMAN]	7.08	2	2	2	1.152	**3.213**	1.574
Q9NPR5	PTPRJ, protein tyrosine phosphatase receptor J, eta (Fragment) OS=Homo sapiens PE=2 SV=1 - [Q9NPR5_HUMAN]	7.76	2	2	2	0.778	**2.002**	1.131
Q6N093	Putative uncharacterized protein DKFZp686I04196 (Fragment) OS=Homo sapiens GN=DKFZp686I04196 PE=2 SV=1 - [Q6N093_HUMAN]	32.37	3	11	20	**0.300**	1.067	1.844
Q6N092	Putative uncharacterized protein DKFZp686K18196 (Fragment) OS=Homo sapiens GN=DKFZp686K18196 PE=2 SV=1 - [Q6N092_HUMAN]	38.15	9	14	18	**0.454**	1.227	**2.330**
Q5CZ94	Putative uncharacterized protein DKFZp781M0386 OS=Homo sapiens GN=DKFZp781M0386 PE=2 SV=1 - [Q5CZ94_HUMAN]	29.91	3	6	12	0.546	1.490	1.292
A0A087WWP7	Receptor-type tyrosine-protein phosphatase gamma OS=Homo sapiens GN=PTPRG PE=1 SV=1 - [A0A087WWP7_HUMAN]	3.18	4	4	4	0.802	**2.102**	1.241
Q15493	Regucalcin OS=Homo sapiens GN=RGN PE=1 SV=1 - [RGN_HUMAN]	11.04	3	3	3	0.604	1.583	1.114
Q96D15	Reticulocalbin-3 OS=Homo sapiens GN=RCN3 PE=1 SV=1 - [RCN3_HUMAN]	8.84	2	2	2	0.829	1.703	1.077
A0A0C4DGV7	Retinol-binding protein 4 OS=Homo sapiens GN=RBP4 PE=1 SV=1 - [A0A0C4DGV7_HUMAN]	47.74	6	6	9	**0.441**	**2.469**	0.902
F5GZZ9	Scavenger receptor cysteine-rich type 1 protein M130 OS=Homo sapiens GN=CD163 PE=1 SV=1 - [F5GZZ9_HUMAN]	8.57	8	8	9	0.699	**2.307**	1.365
O75326	Semaphorin-7A OS=Homo sapiens GN=SEMA7A PE=1 SV=1 - [SEM7A_HUMAN]	3.15	2	2	3	0.878	1.959	1.169
F2RM35	Serine protease OS=Homo sapiens GN=factor IX F9 PE=2 SV=1 - [F2RM35_HUMAN]	4.34	2	2	2	1.338	**3.685**	1.485
Q5UGI6	Serine/cysteine proteinase inhibitor clade G member 1 splice variant 2 (Fragment) OS=Homo sapiens GN=SERPING1 PE=2 SV=1 - [Q5UGI6_HUMAN]	21.62	7	7	9	0.564	**2.910**	1.256
C9J7N5	Serpin I2 (Fragment) OS=Homo sapiens GN=SERPINI2 PE=1 SV=1 - [C9J7N5_HUMAN]	8.25	2	2	2	0.636	**2.325**	1.501
P02743	Serum amyloid P-component OS=Homo sapiens GN=APCS PE=1 SV=2 - [SAMP_HUMAN]	14.35	3	3	5	0.960	**3.509**	1.628
Q5T123	SH3 domain-binding glutamic acid-rich-like protein 3 OS=Homo sapiens GN=SH3BGRL3 PE=1 SV=1 - [Q5T123_HUMAN]	36.36	3	3	5	0.673	1.330	1.030
Q5TFQ8	Signal-regulatory protein beta-1 isoform 3 OS=Homo sapiens GN=SIRPB1 PE=1 SV=1 - [SIRBL_HUMAN]	7.29	2	2	2	0.717	**2.134**	1.254
P09486	SPARC OS=Homo sapiens GN=SPARC PE=1 SV=1 - [SPRC_HUMAN]	40.59	14	14	23	**3.167**	**8.240**	**3.885**
H7BYH4	Superoxide dismutase [Cu-Zn] OS=Homo sapiens GN=SOD1 PE=1 SV=1 - [H7BYH4_HUMAN]	20.00	3	3	4	0.980	1.660	1.234
Q6UWP8	Suprabasin OS=Homo sapiens GN=SBSN PE=1 SV=2 - [SBSN_HUMAN]	15.25	2	2	4	0.641	0.672	1.596
E9PHK0	Tetranectin OS=Homo sapiens GN=CLEC3B PE=1 SV=1 - [E9PHK0_HUMAN]	28.75	3	3	4	1.002	**2.990**	1.928
P62328	Thymosin beta-4 OS=Homo sapiens GN=TMSB4X PE=1 SV=2 - [TYB4_HUMAN]	31.82	3	3	4	1.021	1.878	1.066
P05543	Thyroxine-binding globulin OS=Homo sapiens GN=SERPINA7 PE=1 SV=2 - [THBG_HUMAN]	40.96	15	15	37	0.749	**2.168**	1.256
D3DPF9	Titin, isoform CRA_b OS=Homo sapiens GN=TTN PE=4 SV=1 - [D3DPF9_HUMAN]	0.05	2	2	3	0.600	**2.428**	1.158
J3KQ45	Trans-Golgi network integral membrane protein 2 OS=Homo sapiens GN=TGOLN2 PE=1 SV=1 - [J3KQ45_HUMAN]	9.91	4	4	4	0.988	**3.136**	0.998
P37837	Transaldolase OS=Homo sapiens GN=TALDO1 PE=1 SV=2 - [TALDO_HUMAN]	6.23	2	2	2	0.662	1.655	0.946
P02766	Transthyretin OS=Homo sapiens GN=TTR PE=1 SV=1 - [TTHY_HUMAN]	77.55	10	10	55	0.850	1.780	1.080
Q53HE2	Triosephosphate isomerase (Fragment) OS=Homo sapiens PE=2 SV=1 - [Q53HE2_HUMAN]	28.11	6	6	9	0.607	1.369	0.953
Q9GZX9	Twisted gastrulation protein homolog 1 OS=Homo sapiens GN=TWSG1 PE=1 SV=1 - [TWSG1_HUMAN]	8.52	2	2	2	1.145	**4.876**	1.285
B4E1Z4	Uncharacterized protein OS=Homo sapiens PE=1 SV=1 - [B4E1Z4_HUMAN]	26.54	16	31	62	0.614	**2.176**	1.088
Q8NEJ1	Uncharacterized protein OS=Homo sapiens PE=2 SV=1 - [Q8NEJ1_HUMAN]	38.56	2	9	18	0.693	1.386	0.892
F8UU18	Vascular cell adhesion molecule 1 (Fragment) OS=Homo sapiens GN=VCAM1 PE=4 SV=1 - [F8UU18_HUMAN]	10.26	2	2	2	**0.457**	**2.392**	1.053
Q59F24	Vascular endothelial growth factor receptor 3 variant (Fragment) OS=Homo sapiens PE=2 SV=1 - [Q59F24_HUMAN]	3.70	2	2	3	0.771	**3.404**	1.555
B0YJC4	Vimentin OS=Homo sapiens GN=VIM PE=1 SV=1 - [B0YJC4_HUMAN]	9.05	3	4	5	**5.656**	**7.561**	**4.993**
Q5JQ13	Vinculin (Fragment) OS=Homo sapiens GN=VCL PE=1 SV=1 - [Q5JQ13_HUMAN]	2.23	2	2	2	0.878	**2.412**	1.051
D6RF35	Vitamin D-binding protein OS=Homo sapiens GN=GC PE=1 SV=1 - [D6RF35_HUMAN]	74.37	2	41	245	0.702	1.881	1.147
P02774	Vitamin D-binding protein OS=Homo sapiens GN=GC PE=1 SV=1 - [VTDB_HUMAN]	79.96	3	42	242	0.726	1.954	1.111
P04004	Vitronectin OS=Homo sapiens GN=VTN PE=1 SV=1 - [VTNC_HUMAN]	10.46	5	5	7	1.007	**2.874**	1.134

P54289	Voltage-dependent calcium channel subunit alpha-2/delta-1 OS=Homo sapiens GN=CACNA2D1 PE=1 SV=3 - [CA2D1_HUMAN]	13.15	12	12	13	0.746	**2.146**	1.219
Q2M389	WASH complex subunit 7 OS=Homo sapiens GN=KIAA1033 PE=1 SV=2 - [WASH7_HUMAN]	0.85	2	2	2	0.660	**2.016**	0.670
P25311	Zinc-alpha-2-glycoprotein OS=Homo sapiens GN=AZGP1 PE=1 SV=2 - [ZA2G_HUMAN]	58.39	22	22	84	0.772	1.775	1.034

Supplementary Table 3C

Accession	Description	Coverage	Unique Peptides	Peptides	PSMs	P OP9/Ctrl	S OP9/Ctrl	T OP9/Ctrl
P62261	14-3-3 protein epsilon OS=Bos taurus GN=YWHAE PE=2 SV=1 - [1433E_BOVIN]	22.35	3	6	7	1.516	2.865	2.141
A7Z057	14-3-3 protein gamma OS=Bos taurus GN=YWHAG PE=1 SV=1 - [A7Z057_BOVIN]	26.72	3	7	8	**3.814**	**5.661**	**5.400**
P63103	14-3-3 protein zeta/delta OS=Bos taurus GN=YWHAZ PE=1 SV=1 - [1433Z_BOVIN]	18.37	2	5	6	1.162	3.505	1.028
P82915	28S ribosomal protein S16, mitochondrial OS=Bos taurus GN=MRPS16 PE=1 SV=2 - [RT16_BOVIN]	20.74	2	2	2	0.762	1.657	0.989
Q5EA20	4-hydroxyphenylpyruvate dioxygenase OS=Bos taurus GN=HPD PE=2 SV=3 - [HPPD_BOVIN]	6.36	2	2	2	0.835	1.470	0.975
Q95M17	Acidic mammalian chitinase OS=Bos taurus GN=CHIA PE=1 SV=1 - [CHIA_BOVIN]	17.80	7	7	13	0.732	2.694	1.569
F1MRD0	Actin, cytoplasmic 1 OS=Bos taurus GN=ACTB PE=3 SV=2 - [F1MRD0_BOVIN]	8.27	3	3	6	0.985	4.595	1.301
P07107	Acyl-CoA-binding protein OS=Bos taurus GN=DBI PE=1 SV=2 - [ACBP_BOVIN]	36.78	3	3	3	0.783	2.252	1.123
Q3Y5Z3	Adiponectin OS=Bos taurus GN=ADIPOQ PE=1 SV=1 - [ADIPO_BOVIN]	25.42	5	5	11	0.701	2.195	1.299
Q6XL68	Aggrecan OS=Bos taurus PE=4 SV=1 - [Q6XL68_BOVIN]	3.07	6	6	6	0.881	1.978	1.060
B0JYQ0	ALB protein OS=Bos taurus GN=ALB PE=2 SV=1 - [B0JYQ0_BOVIN]	85.67	2	83	1360	**0.442**	**3.376**	1.010
Q5EA79	Aldose 1-epimerase OS=Bos taurus GN=GALM PE=2 SV=1 - [GALM_BOVIN]	6.14	2	2	3	0.801	3.315	1.218
P09487	Alkaline phosphatase, tissue-nonspecific isozyme OS=Bos taurus GN=ALPL PE=1 SV=2 - [PPBT_BOVIN]	4.20	2	2	2	0.697	1.594	1.091
Q28133	Allergen Bos d 2 OS=Bos taurus PE=1 SV=1 - [ALL2_BOVIN]	36.63	6	6	7	0.796	1.886	1.127
Q3SZR3	Alpha-1-acid glycoprotein OS=Bos taurus GN=ORM1 PE=2 SV=1 - [A1AG_BOVIN]	68.81	19	19	325	0.889	1.685	1.036
P34955	Alpha-1-antiproteinase OS=Bos taurus GN=SERPINA1 PE=1 SV=1 - [A1AT_BOVIN]	62.50	33	33	580	0.922	**2.281**	1.247
Q2KJF1	Alpha-1B-glycoprotein OS=Bos taurus GN=A1BG PE=1 SV=1 - [A1BG_BOVIN]	59.44	20	20	105	0.747	**2.355**	1.186
P28800	Alpha-2-antiplasmin OS=Bos taurus GN=SERPINF2 PE=1 SV=2 - [A2AP_BOVIN]	33.54	19	19	57	0.870	2.279	1.420
P12763	Alpha-2-HS-glycoprotein OS=Bos taurus GN=AHSG PE=1 SV=2 - [FETUA_BOVIN]	49.86	22	22	279	0.999	**3.218**	1.287
Q7SIH1	Alpha-2-macroglobulin OS=Bos taurus GN=A2M PE=1 SV=2 - [A2MG_BOVIN]	27.68	7	33	47	0.603	1.941	1.896
F1MJQ3	Alpha-amylase OS=Bos taurus GN=AMY2B PE=3 SV=1 - [F1MJQ3_BOVIN]	30.53	13	13	18	1.374	3.838	2.261
Q3SZ57	Alpha-fetoprotein OS=Bos taurus GN=AFP PE=2 SV=1 - [FETA_BOVIN]	76.07	42	43	170	**0.190**	0.644	**0.413**
P02662	Alpha-S1-casein OS=Bos taurus GN=CSN1S1 PE=1 SV=2 - [CASA1_BOVIN]	40.65	8	8	9	0.708	11.800	0.695
P02663	Alpha-S2-casein OS=Bos taurus GN=CSN1S2 PE=1 SV=2 - [CASA2_BOVIN]	9.01	2	2	2	1.069	3.786	1.320
E1BC10	Amine oxidase OS=Bos taurus GN=AOC3 PE=1 SV=1 - [E1BC10_BOVIN]	24.12	4	16	76	0.816	**2.888**	1.134
E1BJN3	Amine oxidase OS=Bos taurus PE=3 SV=1 - [E1BJN3_BOVIN]	35.67	10	17	89	0.735	**2.147**	1.164
F1MN84	Aminopeptidase N OS=Bos taurus GN=ANPEP PE=4 SV=2 - [F1MN84_BOVIN]	15.54	15	15	17	0.714	2.026	1.182
Q2KJB3	Angiopoietin-like 3 OS=Bos taurus GN=ANGPTL3 PE=2 SV=1 - [Q2KJB3_BOVIN]	6.32	2	2	3	0.656	2.661	1.099
F1MQJ0	Angiotensin-converting enzyme OS=Bos taurus GN=ACE PE=3 SV=2 - [F1MQJ0_BOVIN]	9.95	12	12	12	0.694	2.109	1.235
Q3SZH5	Angiotensinogen OS=Bos taurus GN=AGT PE=2 SV=1 - [Q3SZH5_BOVIN]	10.85	3	3	4	1.071	1.958	2.588
F1MSZ6	Antithrombin-III OS=Bos taurus GN=SERPINC1 PE=1 SV=1 - [F1MSZ6_BOVIN]	62.37	34	34	270	0.667	1.915	1.157
P15497	Apolipoprotein A-I OS=Bos taurus GN=APOA1 PE=1 SV=3 - [APOA1_BOVIN]	61.89	18	18	31	0.694	2.529	1.384
P81644	Apolipoprotein A-II OS=Bos taurus GN=APOA2 PE=1 SV=2 - [APOA2_BOVIN]	21.00	3	3	3	0.493	1.567	0.862
F1N3Q7	Apolipoprotein A-IV OS=Bos taurus GN=APOA4 PE=3 SV=1 - [F1N3Q7_BOVIN]	71.32	31	31	82	0.900	**3.232**	1.600
P19035	Apolipoprotein C-III OS=Bos taurus GN=APOC3 PE=1 SV=2 - [APOC3_BOVIN]	22.92	2	2	3	0.449	2.237	0.869
F1MS32	Apolipoprotein D OS=Bos taurus GN=APOD PE=3 SV=2 - [F1MS32_BOVIN]	20.11	3	3	3	1.000	2.883	1.779
Q0ZCB4	Apolipoprotein E (Fragment) OS=Bos taurus PE=3 SV=1 - [Q0ZCB4_BOVIN]	10.55	3	3	4	0.820	1.665	1.059

Q2KIH2	ApoN protein OS=Bos taurus GN=ApoN PE=2 SV=1 - [Q2KIH2_BOVIN]	12.20	3	3	4	0.899	2.588	1.417
P33097	Aspartate aminotransferase, cytoplasmic OS=Bos taurus GN=GOT1 PE=1 SV=3 - [AATC_BOVIN]	20.58	7	7	8	0.808	2.858	1.402
Q1RMN0	Aspartylglucosaminidase OS=Bos taurus GN=AGA PE=2 SV=1 - [Q1RMN0_BOVIN]	6.40	2	2	2	0.811	1.903	1.092
F1MLB8	ATP synthase subunit alpha OS=Bos taurus GN=ATP5A1 PE=1 SV=1 - [F1MLB8_BOVIN]	3.62	2	2	2	0.440	1.903	1.145
Q8MJ16	Attractin OS=Bos taurus GN=ATRN PE=2 SV=1 - [Q8MJ16_BOVIN]	24.10	31	32	58	0.758	2.075	1.219
Q862Q3	Beta-2-microglobulin (Fragment) OS=Bos taurus PE=2 SV=1 - [Q862Q3_BOVIN]	79.57	7	7	12	0.847	2.132	1.055
P02666	Beta-casein OS=Bos taurus GN=CSN2 PE=1 SV=2 - [CASB_BOVIN]	15.18	3	3	4	0.911	9.058	0.701
A6QQ07	Biotinidase OS=Bos taurus GN=BTD PE=2 SV=1 - [BTD_BOVIN]	19.43	9	9	10	0.975	2.903	1.652
A6QPP9	C-X-C motif chemokine OS=Bos taurus GN=PF4 PE=2 SV=1 - [A6QPP9_BOVIN]	40.68	6	6	17	0.605	1.538	1.035
F1N619	Cadherin-1 OS=Bos taurus GN=CDH1 PE=1 SV=2 - [F1N619_BOVIN]	1.57	2	2	2	0.825	1.840	1.122
F1MKP6	Cadherin-13 OS=Bos taurus GN=CDH13 PE=4 SV=2 - [F1MKP6_BOVIN]	16.59	9	9	10	1.022	2.007	0.954
P19534	Cadherin-2 (Fragment) OS=Bos taurus GN=CDH2 PE=2 SV=1 - [CADH2_BOVIN]	4.56	2	2	2	0.615	1.475	1.520
G5E588	Cadherin-5 OS=Bos taurus GN=CDH5 PE=4 SV=1 - [G5E588_BOVIN]	6.14	4	4	5	0.704	3.226	1.262
P62157	Calmodulin OS=Bos taurus GN=CALM PE=1 SV=2 - [CALM_BOVIN]	18.79	3	3	3	1.714	2.567	1.761
A5D7J6	CALR protein OS=Bos taurus GN=CALR PE=1 SV=1 - [A5D7J6_BOVIN]	25.66	10	10	11	0.977	3.239	2.008
F1N3H1	Calumenin OS=Bos taurus GN=CALU PE=1 SV=1 - [F1N3H1_BOVIN]	12.70	2	2	2	1.246	13.400	7.715
P00921	Carbonic anhydrase 2 OS=Bos taurus GN=CA2 PE=1 SV=3 - [CAH2_BOVIN]	20.77	4	4	4	0.803	2.350	1.126
A0A140T835	Carboxylic ester hydrolase OS=Bos taurus GN=ACHE PE=3 SV=1 - [A0A140T835_BOVIN]	3.43	2	2	2	0.791	2.091	1.345
Q2KIG3	Carboxypeptidase B2 OS=Bos taurus GN=CPB2 PE=1 SV=1 - [CBPB2_BOVIN]	5.44	2	2	2	0.395	1.437	0.917
Q17QK3	Carboxypeptidase Q OS=Bos taurus GN=CPQ PE=2 SV=1 - [CBPQ_BOVIN]	22.88	10	10	12	0.758	1.942	1.251
P19660	Cathelicidin-2 OS=Bos taurus GN=CATHL2 PE=1 SV=2 - [CTHL2_BOVIN]	14.20	2	2	3	0.791	1.778	1.115
P07688	Cathepsin B OS=Bos taurus GN=CTSB PE=1 SV=5 - [CATB_BOVIN]	23.28	6	6	9	0.764	1.760	1.269
Q864S1	Cathepsin C (Fragment) OS=Bos taurus PE=2 SV=1 - [Q864S1_BOVIN]	21.40	8	8	9	0.668	1.837	1.013
P25326	Cathepsin S OS=Bos taurus GN=CTSS PE=1 SV=2 - [CATS_BOVIN]	11.18	3	3	3	0.821	3.289	1.620
F1MW68	Cathepsin Z OS=Bos taurus GN=CTSZ PE=3 SV=1 - [F1MW68_BOVIN]	10.20	3	3	3	2.207	8.209	2.966
P08169	Cation-independent mannose-6-phosphate receptor OS=Bos taurus GN=IGF2R PE=1 SV=2 - [MPRI_BOVIN]	10.80	27	27	30	0.695	2.383	1.340
A5PJP4	CBLN2 protein OS=Bos taurus GN=CBLN2 PE=2 SV=1 - [A5PJP4_BOVIN]	6.70	2	2	2	0.881	2.596	1.158
F1MHN8	CD166 antigen OS=Bos taurus GN=ALCAM PE=4 SV=2 - [F1MHN8_BOVIN]	10.63	5	5	5	0.804	1.791	1.050
F1MHC3	CD44 antigen OS=Bos taurus GN=CD44 PE=4 SV=1 - [F1MHC3_BOVIN]	10.11	4	4	11	0.848	1.552	1.038
A7YW37	CD58 protein (Fragment) OS=Bos taurus GN=CD58 PE=2 SV=1 - [A7YW37_BOVIN]	16.15	4	4	4	0.822	1.928	1.153
Q32PA1	CD59 molecule, complement regulatory protein OS=Bos taurus GN=CD59 PE=2 SV=1 - [Q32PA1_BOVIN]	22.31	3	3	4	0.857	2.170	1.234
A6QNW7	CD5L protein OS=Bos taurus GN=CD5L PE=2 SV=1 - [A6QNW7_BOVIN]	39.91	18	18	47	0.753	1.752	1.067
Q9GL33	CD86 antigen (Fragment) OS=Bos taurus GN=CD86 PE=2 SV=1 - [Q9GL33_BOVIN]	6.34	2	2	2	1.079	1.922	1.299
Q2TBL2	Cell adhesion molecule 1 OS=Bos taurus GN=CADM1 PE=2 SV=1 - [Q2TBL2_BOVIN]	21.82	6	6	7	0.827	1.746	1.023
P32749	Cholinesterase OS=Bos taurus GN=BCHE PE=2 SV=2 - [CHLE_BOVIN]	11.79	9	9	13	0.744	2.253	1.215
A0A140T879	Chromogranin-A OS=Bos taurus GN=CHGA PE=1 SV=1 - [A0A140T879_BOVIN]	6.24	2	2	2	1.009	1.342	0.915
P17697	Clusterin OS=Bos taurus GN=CLU PE=1 SV=1 - [CLUS_BOVIN]	22.10	10	10	15	0.741	1.843	1.123
Q2HJ57	Coactosin-like protein OS=Bos taurus GN=COTL1 PE=2 SV=3 - [COTL1_BOVIN]	49.30	6	6	6	0.814	2.723	1.449

P00741	Coagulation factor IX OS=Bos taurus GN=F9 PE=1 SV=2 - [FA9_BOVIN]	13.20	6	6	6	0.811	2.156	1.133
Q28107	Coagulation factor V OS=Bos taurus GN=F5 PE=1 SV=1 - [FA5_BOVIN]	3.08	6	6	7	0.925	1.836	1.088
P02453	Collagen alpha-1(I) chain OS=Bos taurus GN=COL1A1 PE=1 SV=3 - [CO1A1_BOVIN]	9.43	10	11	20	1.000	1.742	1.022
F1MXS8	Collagen alpha-1(III) chain OS=Bos taurus GN=COL3A1 PE=4 SV=2 - [F1MXS8_BOVIN]	6.48	7	8	12	0.863	2.445	1.300
F1N0K0	Collagen alpha-1(XI) chain OS=Bos taurus GN=COL11A1 PE=4 SV=2 - [F1N0K0_BOVIN]	2.31	2	5	5	1.176	3.753	2.027
P02465	Collagen alpha-2(I) chain OS=Bos taurus GN=COL1A2 PE=1 SV=2 - [CO1A2_BOVIN]	3.23	4	4	5	**9.218**	**6.114**	1.000
P42916	Collectin-43 OS=Bos taurus GN=CL43 PE=1 SV=2 - [CL43_BOVIN]	38.63	10	10	14	0.839	1.933	1.048
Q0VCX1	Complement C1s subcomponent OS=Bos taurus GN=C1S PE=2 SV=2 - [C1S_BOVIN]	4.93	3	3	4	0.913	1.683	1.000
A0A0A0MP91	Complement C2 OS=Bos taurus GN=C2 PE=3 SV=1 - [A0A0A0MP91_BOVIN]	23.33	18	18	36	0.726	2.269	1.285
Q2UVX4	Complement C3 OS=Bos taurus GN=C3 PE=1 SV=2 - [CO3_BOVIN]	54.55	85	85	185	0.675	**3.680**	1.576
A5D9E9	Complement component 1, r subcomponent OS=Bos taurus GN=C1R PE=2 SV=1 - [A5D9E9_BOVIN]	3.83	3	3	3	0.900	1.565	1.264
F1MM86	Complement component C6 OS=Bos taurus GN=C6 PE=4 SV=1 - [F1MM86_BOVIN]	3.11	3	3	3	0.309	1.238	0.928
F1N045	Complement component C7 OS=Bos taurus GN=C7 PE=4 SV=1 - [F1N045_BOVIN]	4.86	4	4	4	0.559	1.871	1.289
P81187	Complement factor B OS=Bos taurus GN=CFB PE=1 SV=2 - [CFAB_BOVIN]	56.37	37	38	114	0.634	**2.815**	1.278
F1MVI0	Contactin-1 OS=Bos taurus GN=CNTN1 PE=4 SV=1 - [F1MVI0_BOVIN]	5.30	5	5	5	0.760	1.900	1.166
E1BF81	Corticosteroid-binding globulin OS=Bos taurus GN=SERPINA6 PE=3 SV=1 - [CBG_BOVIN]	32.18	12	12	25	0.748	1.930	1.144
A6QP30	CPN2 protein OS=Bos taurus GN=CPN2 PE=2 SV=1 - [A6QP30_BOVIN]	3.28	2	2	2	0.613	2.007	1.794
B8Y9T0	Cumulus cell-specific fibronectin 1 transcript variant OS=Bos taurus GN=FN1 PE=1 SV=1 - [B8Y9T0_BOVIN]	7.10	14	14	15	0.842	1.613	1.072
Q1RMP3	CutA divalent cation tolerance homolog (E. coli) OS=Bos taurus GN=CUTA PE=2 SV=1 - [Q1RMP3_BOVIN]	18.30	2	2	2	0.721	1.622	1.226
P62894	Cytochrome c OS=Bos taurus GN=CYCS PE=1 SV=2 - [CYC_BOVIN]	56.19	6	6	9	0.916	1.734	1.198
Q45VK8	DAF-2 OS=Bos taurus GN=CD55 PE=2 SV=1 - [Q45VK8_BOVIN]	6.27	2	2	2	0.772	1.976	1.322
E1BIR2	Dipeptidase OS=Bos taurus GN=DPEP2 PE=3 SV=1 - [E1BIR2_BOVIN]	5.58	3	3	3	0.721	2.383	1.686
P81425	Dipeptidyl peptidase 4 OS=Bos taurus GN=DPP4 PE=1 SV=3 - [DPP4_BOVIN]	24.58	17	17	19	0.717	2.157	1.162
A6QL81	DKK3 protein OS=Bos taurus GN=DKK3 PE=2 SV=1 - [A6QL81_BOVIN]	12.32	3	3	3	0.967	1.347	1.033
A2VE41	EGF-containing fibulin-like extracellular matrix protein 1 OS=Bos taurus GN=EFEMP1 PE=1 SV=1 - [A2VE41_BOV	14.86	6	6	8	0.869	1.212	1.000
Q28105	Endothelial protein C receptor OS=Bos taurus GN=PROCR PE=2 SV=1 - [EPCR_BOVIN]	14.94	3	3	6	0.769	1.914	1.442
Q3MHW2	F10 protein (Fragment) OS=Bos taurus GN=F10 PE=2 SV=1 - [Q3MHW2_BOVIN]	12.63	6	6	6	0.920	2.538	1.676
F1MHQ4	Fatty acid-binding protein, adipocyte OS=Bos taurus GN=FABP4 PE=1 SV=2 - [F1MHQ4_BOVIN]	14.39	2	2	2	**8.713**	**24.442**	**9.778**
P80425	Fatty acid-binding protein, liver OS=Bos taurus GN=FABP1 PE=1 SV=1 - [FABPL_BOVIN]	14.17	2	2	4	0.726	1.867	1.260
Q58D62	Fetuin-B OS=Bos taurus GN=FETUB PE=1 SV=1 - [FETUB_BOVIN]	54.26	21	21	96	0.953	**3.517**	1.520
A5PJE3	Fibrinogen alpha chain OS=Bos taurus GN=FGA PE=1 SV=1 - [A5PJE3_BOVIN]	19.19	10	10	15	0.575	1.328	0.914
A4IFL5	Fibroblast growth factor receptor OS=Bos taurus GN=FGFR1 PE=2 SV=2 - [A4IFL5_BOVIN]	3.05	2	2	3	0.843	1.000	1.023
Q29RY7	Fibroleukin OS=Bos taurus GN=FGL2 PE=2 SV=1 - [FGL2_BOVIN]	14.74	8	8	12	0.949	2.192	1.175
A5D7S8	Fibulin-1 OS=Bos taurus GN=FBLN1 PE=2 SV=1 - [A5D7S8_BOVIN]	8.22	6	6	7	0.723	1.182	0.935
P02702	Folate receptor alpha OS=Bos taurus GN=FOLR1 PE=1 SV=3 - [FOLR1_BOVIN]	16.60	4	4	4	0.903	2.377	1.040
Q58D84	Follistatin-related protein 1 OS=Bos taurus GN=FSTL1 PE=2 SV=1 - [FSTL1_BOVIN]	17.26	6	6	7	**2.697**	**8.102**	**3.193**
A7E3W2	Galectin-3-binding protein OS=Bos taurus GN=LGALS3BP PE=1 SV=1 - [LG3BP_BOVIN]	19.64	9	9	15	0.613	1.809	1.131
A6H7H5	Gamma-tubulin complex component OS=Bos taurus GN=TUBGCP5 PE=2 SV=1 - [A6H7H5_BOVIN]	2.25	2	2	2	2.035	1.866	1.059

F1MJH1	Gelsolin OS=Bos taurus GN=GSN PE=1 SV=1 - [F1MJH1_BOVIN]	7.39	5	5	6	0.661	2.073	1.105
Q3ZBD7	Glucose-6-phosphate isomerase OS=Bos taurus GN=GPI PE=2 SV=4 - [G6PI_BOVIN]	23.16	11	11	12	0.737	2.393	1.178
F1MEM5	Glutamyl aminopeptidase OS=Bos taurus GN=ENPEP PE=4 SV=1 - [F1MEM5_BOVIN]	3.97	4	4	4	1.100	2.204	1.214
G3X8D7	Glutathione peroxidase OS=Bos taurus GN=GPX3 PE=3 SV=1 - [G3X8D7_BOVIN]	8.48	2	2	3	0.303	0.526	0.380
P02081	Hemoglobin fetal subunit beta OS=Bos taurus PE=1 SV=1 - [HBBF_BOVIN]	71.03	7	10	14	0.608	1.908	1.199
P01966	Hemoglobin subunit alpha OS=Bos taurus GN=HBA PE=1 SV=2 - [HBA_BOVIN]	81.69	8	8	17	0.609	1.550	0.894
P02070	Hemoglobin subunit beta OS=Bos taurus GN=HBB PE=1 SV=1 - [HBB_BOVIN]	75.86	8	11	26	0.629	1.404	0.900
Q3SZV7	Hemopexin OS=Bos taurus GN=HPX PE=2 SV=1 - [HEMO_BOVIN]	64.49	29	29	270	0.906	**2.886**	1.382
Q3SYR8	Immunoglobulin J chain OS=Bos taurus GN=IGJ PE=1 SV=1 - [Q3SYR8_BOVIN]	33.76	5	5	10	0.855	1.642	1.119
I7CLV3	Insulin (Fragment) OS=Bos taurus PE=2 SV=1 - [I7CLV3_BOVIN]	35.80	2	2	11	0.736	1.557	1.075
P53712	Integrin beta-1 OS=Bos taurus GN=ITGB1 PE=1 SV=3 - [ITB1_BOVIN]	3.38	3	3	4	0.727	2.467	1.415
F1MMP5	Inter-alpha-trypsin inhibitor heavy chain H1 OS=Bos taurus GN=ITIH1 PE=4 SV=1 - [F1MMP5_BOVIN]	3.31	2	2	2	0.502	2.609	0.828
P56652	Inter-alpha-trypsin inhibitor heavy chain H3 OS=Bos taurus GN=ITIH3 PE=1 SV=2 - [ITIH3_BOVIN]	5.05	5	5	5	0.785	2.741	1.800
F1MMD7	Inter-alpha-trypsin inhibitor heavy chain H4 OS=Bos taurus GN=ITIH4 PE=1 SV=2 - [F1MMD7_BOVIN]	21.83	17	17	23	0.777	3.143	1.788
F1MI38	Intercellular adhesion molecule 1 OS=Bos taurus GN=ICAM1 PE=4 SV=1 - [F1MI38_BOVIN]	9.53	5	5	7	0.876	2.301	1.288
Q0VC51	Interleukin 1 receptor accessory protein OS=Bos taurus GN=IL1RAP PE=2 SV=1 - [Q0VC51_BOVIN]	38.06	14	14	24	1.269	2.967	1.510
A5D7R6	ITIH2 protein OS=Bos taurus GN=ITIH2 PE=2 SV=1 - [A5D7R6_BOVIN]	6.87	8	8	9	0.692	2.113	1.249
F1MNV5	Kininogen-1 OS=Bos taurus GN=KNG1 PE=1 SV=2 - [F1MNV5_BOVIN]	68.35	10	32	110	0.813	**3.385**	1.435
P01045	Kininogen-2 OS=Bos taurus GN=KNG2 PE=1 SV=1 - [KNG2_BOVIN]	47.33	9	31	114	0.886	**2.522**	1.429
B0JYN3	L-lactate dehydrogenase OS=Bos taurus GN=LDHB PE=2 SV=1 - [B0JYN3_BOVIN]	6.89	2	2	2	0.630	1.572	0.756
Q2KIW4	Lecithin-cholesterol acyltransferase OS=Bos taurus GN=LCAT PE=2 SV=1 - [Q2KIW4_BOVIN]	7.50	3	3	3	0.647	2.380	1.960
A0A140T8D4	Legumain OS=Bos taurus GN=LGMN PE=4 SV=1 - [A0A140T8D4_BOVIN]	22.86	10	10	13	0.645	2.574	1.203
Q2KIF2	Leucine-rich alpha-2-glycoprotein 1 OS=Bos taurus GN=LRG1 PE=2 SV=1 - [Q2KIF2_BOVIN]	50.58	18	18	77	0.845	2.221	1.325
A7Z077	LOC510651 protein OS=Bos taurus GN=LOC510651 PE=2 SV=1 - [A7Z077_BOVIN]	0.90	2	2	2	0.662	1.985	0.800
Q05443	Lumican OS=Bos taurus GN=LUM PE=1 SV=1 - [LUM_BOVIN]	46.49	12	12	31	0.894	2.560	1.628
Q05204	Lysosome-associated membrane glycoprotein 1 OS=Bos taurus GN=LAMP1 PE=1 SV=2 - [LAMP1_BOVIN]	9.05	4	4	8	0.828	1.735	1.115
B5B0D4	Major allergen beta-lactoglobulin OS=Bos taurus PE=2 SV=1 - [B5B0D4_BOVIN]	16.29	2	2	2	1.185	1.541	1.035
Q1JP81	Major histocompatibility complex, class II, DR beta 4 OS=Bos taurus GN=HLA-DRB4 PE=2 SV=1 - [Q1JP81_BOVI	7.89	2	2	2	0.770	1.681	0.977
P43481	Mast/stem cell growth factor receptor Kit OS=Bos taurus GN=KIT PE=2 SV=1 - [KIT_BOVIN]	2.35	3	3	3	0.642	2.187	1.237
Q9GMB4	Membrane tyrosine phosphatase (Fragment) OS=Bos taurus GN=cd45 PE=2 SV=1 - [Q9GMB4_BOVIN]	3.25	2	2	2	0.812	1.741	1.288
Q6GVI3	MHC class II antigen (Fragment) OS=Bos taurus GN=BoLA-DRA PE=4 SV=1 - [Q6GVI3_BOVIN]	29.00	2	2	2	0.829	1.746	1.099
P02192	Myoglobin OS=Bos taurus GN=MB PE=1 SV=3 - [MYG_BOVIN]	20.13	3	3	3	0.573	1.426	0.920
Q9BE40	Myosin-1 OS=Bos taurus GN=MYH1 PE=2 SV=2 - [MYH1_BOVIN]	1.29	3	3	3	0.797	3.044	0.847
Q95KW3	NAD(P)(+)--arginine ADP-ribosyltransferase (Fragment) OS=Bos taurus GN=ART3 PE=2 SV=1 - [Q95KW3_BOVI	8.26	2	2	2	0.608	2.035	1.449
F1N1W7	Neural cell adhesion molecule 1 OS=Bos taurus GN=NCAM1 PE=4 SV=2 - [F1N1W7_BOVIN]	10.91	7	7	11	0.755	2.772	1.376
Q0P569	Nucleobindin-1 OS=Bos taurus GN=NUCB1 PE=2 SV=1 - [NUCB1_BOVIN]	27.43	11	11	14	**4.032**	**16.142**	**4.954**
Q17QF1	Oncostatin M receptor OS=Bos taurus GN=OSMR PE=2 SV=1 - [Q17QF1_BOVIN]	33.02	2	9	17	0.830	1.669	1.148
G3X6Y4	Osteomodulin OS=Bos taurus GN=OMD PE=4 SV=1 - [G3X6Y4_BOVIN]	15.88	6	6	9	0.914	2.020	1.308

Q2QCT5	Osteopontin (Fragment) OS=Bos taurus PE=2 SV=1 - [Q2QCT5_BOVIN]	16.09	5	5	5	0.835	1.562	0.978
C4T8B4	Pentaxin OS=Bos taurus GN=CRP PE=2 SV=1 - [C4T8B4_BOVIN]	8.93	2	2	2	0.858	2.259	1.358
Q58D34	Peptidase inhibitor 16 OS=Bos taurus GN=PI16 PE=2 SV=1 - [PI16_BOVIN]	8.19	3	3	5	0.775	2.419	1.129
P80109	Phosphatidylinositol-glycan-specific phospholipase D OS=Bos taurus GN=GPLD1 PE=1 SV=1 - [PHLD_BOVIN]	2.62	2	2	2	0.844	3.654	1.453
F1MDS0	Phospholipid phosphatase-related protein type 2 OS=Bos taurus GN=PRG4 PE=4 SV=2 - [F1MDS0_BOVIN]	27.67	20	20	47	0.840	1.699	1.026
F1MX49	Platelet-derived growth factor receptor alpha OS=Bos taurus GN=PDGFRA PE=3 SV=2 - [F1MX49_BOVIN]	2.02	2	2	2	**2.894**	**2.600**	1.814
P21782	Pleiotrophin OS=Bos taurus GN=PTN PE=1 SV=2 - [PTN_BOVIN]	9.52	2	2	2	**2.918**	**8.592**	**2.889**
A7E350	PLG protein OS=Bos taurus GN=PLG PE=2 SV=1 - [A7E350_BOVIN]	15.47	11	11	21	0.658	1.353	0.912
Q8I019	Poliovirus receptor related-gene 1 (Fragment) OS=Bos taurus GN=prr1 PE=4 SV=1 - [Q8I019_BOVIN]	22.45	2	2	2	0.869	3.661	1.276
A9UIB1	PON1 OS=Bos taurus GN=PON1 PE=2 SV=1 - [A9UIB1_BOVIN]	7.61	3	3	4	1.100	1.818	1.401
Q29437	Primary amine oxidase, liver isozyme OS=Bos taurus PE=1 SV=1 - [AOCX_BOVIN]	49.74	16	27	194	0.707	**2.052**	1.214
A5D7B7	Prolyl endopeptidase FAP OS=Bos taurus GN=FAP PE=1 SV=1 - [SEPR_BOVIN]	2.37	2	2	2	0.842	2.720	1.136
A1L555	Prosaposin OS=Bos taurus GN=PSAP PE=2 SV=1 - [A1L555_BOVIN]	6.67	4	4	5	1.077	2.651	1.757
G5E5C3	Proteasome subunit alpha type OS=Bos taurus GN=PSMA6 PE=3 SV=1 - [G5E5C3_BOVIN]	9.39	2	2	2	1.512	2.245	2.740
Q58DU5	Proteasome subunit alpha type-3 OS=Bos taurus GN=PSMA3 PE=1 SV=3 - [PSA3_BOVIN]	8.63	2	2	2	0.853	1.916	1.262
F1MMK9	Protein AMBP OS=Bos taurus GN=AMBP PE=4 SV=2 - [F1MMK9_BOVIN]	41.48	14	14	34	1.324	3.827	1.605
Q5E946	Protein deglycase DJ-1 OS=Bos taurus GN=PARK7 PE=2 SV=1 - [PARK7_BOVIN]	26.98	4	4	4	0.608	1.799	1.071
A6H7J6	Protein disulfide-isomerase OS=Bos taurus GN=P4HB PE=1 SV=1 - [A6H7J6_BOVIN]	13.53	7	7	7	0.944	2.527	1.500
A5D7E8	Protein disulfide-isomerase OS=Bos taurus GN=PDIA3 PE=1 SV=1 - [A5D7E8_BOVIN]	6.34	3	3	3	0.616	4.341	1.511
Q2KIT0	Protein HP-20 homolog OS=Bos taurus PE=2 SV=1 - [HP20_BOVIN]	54.97	11	11	48	0.691	2.684	1.244
Q2KIX7	Protein HP-25 homolog 1 OS=Bos taurus PE=1 SV=1 - [HP251_BOVIN]	30.66	6	7	24	0.810	2.465	1.107
Q2KIU3	Protein HP-25 homolog 2 OS=Bos taurus PE=2 SV=1 - [HP252_BOVIN]	43.72	8	9	34	0.778	2.348	1.146
P35466	Protein S100-A4 OS=Bos taurus GN=S100A4 PE=1 SV=2 - [S10A4_BOVIN]	15.84	2	2	2	1.188	2.666	1.572
P00735	Prothrombin OS=Bos taurus GN=F2 PE=1 SV=2 - [THRB_BOVIN]	37.60	22	22	102	0.936	1.883	1.078
A5D7Q2	Putative uncharacterized protein OS=Bos taurus PE=2 SV=1 - [A5D7Q2_BOVIN]	6.16	2	2	2	1.005	2.468	1.145
A5PK72	Putative uncharacterized protein OS=Bos taurus PE=2 SV=1 - [A5PK72_BOVIN]	42.80	2	9	27	0.553	1.331	0.813
A6QM09	Putative uncharacterized protein OS=Bos taurus PE=2 SV=1 - [A6QM09_BOVIN]	59.48	5	12	29	0.782	2.026	1.023
E0AE18	Receptor protein-tyrosine kinase OS=Bos taurus GN=EGFR PE=2 SV=1 - [E0AE18_BOVIN]	6.75	5	6	7	0.750	1.154	1.342
Q9TTJ5	Regucalcin OS=Bos taurus GN=RGN PE=2 SV=1 - [RGN_BOVIN]	23.08	6	6	6	0.413	1.355	0.864
Q2KJ39	Reticulocalbin-3 OS=Bos taurus GN=RCN3 PE=2 SV=1 - [RCN3_BOVIN]	8.84	2	2	2	0.829	1.703	1.077
P18902	Retinol-binding protein 4 OS=Bos taurus GN=RBP4 PE=1 SV=1 - [RET4_BOVIN]	37.70	6	6	12	0.445	2.255	0.893
P85521	Scavenger receptor cysteine-rich type 1 protein M130 OS=Bos taurus GN=CD163 PE=1 SV=2 - [C163A_BOVIN]	8.15	9	9	9	0.768	2.237	1.495
G3X6N3	Serotransferrin OS=Bos taurus GN=TF PE=1 SV=1 - [G3X6N3_BOVIN]	93.75	8	104	4606	0.654	**2.063**	1.163
Q29443	Serotransferrin OS=Bos taurus GN=TF PE=2 SV=1 - [TRFE_BOVIN]	93.75	8	104	4634	0.678	**2.018**	1.038
Q2HJF0	Serotransferrin-like OS=Bos taurus GN=LOC525947 PE=2 SV=1 - [Q2HJF0_BOVIN]	36.98	14	18	167	0.724	**4.100**	1.305
Q9TTE1	Serpin A3-1 OS=Bos taurus GN=SERPINA3-1 PE=1 SV=3 - [SPA31_BOVIN]	74.21	2	27	667	0.947	1.429	1.000
A0A0A0MP89	Serpin A3-8 OS=Bos taurus GN=SERPINA3-8 PE=3 SV=1 - [A0A0A0MP89_BOVIN]	53.11	17	22	289	0.787	**2.061**	1.174
Q3SYR0	Serpin peptidase inhibitor, clade A member 7 OS=Bos taurus GN=SERPINA7 PE=2 SV=1 - [Q3SYR0_BOVIN]	46.23	21	21	72	0.713	2.234	1.287

A5PJ69	SERPINA10 protein OS=Bos taurus GN=SERPINA10 PE=2 SV=1 - [A5PJ69_BOVIN]	5.97	3	3	3	0.673	2.348	1.174
A6QPW6	SERPINI2 protein OS=Bos taurus GN=SERPINI2 PE=2 SV=1 - [A6QPW6_BOVIN]	7.65	3	3	4	0.877	2.899	1.574
A0A140T897	Serum albumin OS=Bos taurus GN=ALB PE=1 SV=1 - [A0A140T897_BOVIN]	93.57	11	92	1717	0.704	**3.086**	1.272
E1BJF9	Serum amyloid A protein OS=Bos taurus GN=LOC104968478 PE=3 SV=1 - [E1BJF9_BOVIN]	29.23	4	4	4	0.537	1.084	0.545
Q3T004	Serum amyloid P-component OS=Bos taurus GN=APCS PE=2 SV=1 - [SAMP_BOVIN]	36.61	8	8	11	0.700	2.648	1.253
Q6T182	Sex hormone-binding globulin (Fragment) OS=Bos taurus GN=SHBG PE=2 SV=1 - [Q6T182_BOVIN]	11.32	4	4	6	0.675	1.766	2.136
Q3ZCL8	SH3 domain-binding glutamic acid-rich-like protein 3 OS=Bos taurus GN=SH3BGRL3 PE=3 SV=1 - [SH3L3_BOVIN	34.41	3	3	5	0.673	1.330	1.030
A6QQN6	SMPDL3B protein OS=Bos taurus GN=SMPDL3B PE=2 SV=1 - [A6QQN6_BOVIN]	9.43	4	4	4	0.602	2.432	1.243
P13213	SPARC OS=Bos taurus GN=SPARC PE=1 SV=2 - [SPRC_BOVIN]	40.59	15	15	25	**3.167**	**8.240**	**3.885**
Q3SYW7	SPARC-like 1 (Hevin) OS=Bos taurus GN=SPARCL1 PE=2 SV=1 - [Q3SYW7_BOVIN]	6.42	3	3	3	0.909	1.773	1.206
Q3ZCH0	Stress-70 protein, mitochondrial OS=Bos taurus GN=HSPA9 PE=2 SV=1 - [GRP75_BOVIN]	2.80	2	2	2	0.714	2.060	1.345
A3KLR9	Superoxide dismutase [Cu-Zn] OS=Bos taurus GN=ECSOD PE=1 SV=1 - [A3KLR9_BOVIN]	16.60	3	3	3	0.868	1.268	0.984
P00442	Superoxide dismutase [Cu-Zn] OS=Bos taurus GN=SOD1 PE=1 SV=2 - [SODC_BOVIN]	37.50	6	6	8	0.814	1.710	1.040
Q2KIS7	Tetranectin OS=Bos taurus GN=CLEC3B PE=2 SV=1 - [TETN_BOVIN]	29.70	4	4	6	0.984	3.174	1.928
A6QQ20	TGOLN2 protein (Fragment) OS=Bos taurus GN=TGOLN2 PE=1 SV=1 - [A6QQ20_BOVIN]	20.00	4	4	5	0.985	1.577	1.023
F1N3A1	Thrombospondin-1 OS=Bos taurus GN=THBS1 PE=4 SV=1 - [F1N3A1_BOVIN]	1.71	2	2	2	0.369	1.821	0.572
G5E5C8	Transaldolase OS=Bos taurus GN=TALDO1 PE=1 SV=1 - [G5E5C8_BOVIN]	9.50	3	3	3	0.716	1.750	1.070
O46375	Transthyretin OS=Bos taurus GN=TTR PE=1 SV=1 - [TTHY_BOVIN]	75.51	12	12	111	1.041	1.839	1.012
E1B8N4	Trehalase OS=Bos taurus GN=TREH PE=3 SV=2 - [E1B8N4_BOVIN]	18.65	8	8	10	0.707	1.879	1.202
Q5E956	Triosephosphate isomerase OS=Bos taurus GN=TPI1 PE=2 SV=3 - [TPIS_BOVIN]	44.58	10	10	15	0.603	1.476	0.858
Q0VD44	Twisted gastrulation homolog 1 (Drosophila) OS=Bos taurus GN=TWSG1 PE=2 SV=1 - [Q0VD44_BOVIN]	8.52	2	2	2	1.145	4.876	1.285
G3MYZ3	Uncharacterized protein OS=Bos taurus GN=AFM PE=4 SV=1 - [G3MYZ3_BOVIN]	53.15	33	33	62	0.756	2.315	1.296
E1BL29	Uncharacterized protein OS=Bos taurus GN=BLMH PE=4 SV=1 - [E1BL29_BOVIN]	19.17	7	7	8	0.702	2.137	1.267
F1N7F9	Uncharacterized protein OS=Bos taurus GN=CACNA2D1 PE=4 SV=2 - [F1N7F9_BOVIN]	17.68	15	15	16	0.714	2.120	1.207
F1MPE1	Uncharacterized protein OS=Bos taurus GN=CD109 PE=4 SV=2 - [F1MPE1_BOVIN]	18.59	22	22	29	0.780	2.187	1.296
F1MN25	Uncharacterized protein OS=Bos taurus GN=CD34 PE=4 SV=1 - [F1MN25_BOVIN]	4.19	2	2	2	0.611	2.804	1.145
G3N251	Uncharacterized protein OS=Bos taurus GN=CENPE PE=3 SV=1 - [G3N251_BOVIN]	0.84	2	2	2	0.608	2.420	1.113
F1N4M7	Uncharacterized protein OS=Bos taurus GN=CFI PE=1 SV=2 - [F1N4M7_BOVIN]	4.69	3	3	3	0.814	1.879	1.215
F1MW33	Uncharacterized protein OS=Bos taurus GN=CHL1 PE=4 SV=2 - [F1MW33_BOVIN]	7.17	7	8	8	0.690	2.024	1.204
G5E5W4	Uncharacterized protein OS=Bos taurus GN=CNTNAP4 PE=4 SV=1 - [G5E5W4_BOVIN]	4.11	2	2	2	1.000	1.000	1.000
G3MZI7	Uncharacterized protein OS=Bos taurus GN=COL5A1 PE=4 SV=1 - [G3MZI7_BOVIN]	3.51	4	5	5	0.692	2.381	1.118
E1BI98	Uncharacterized protein OS=Bos taurus GN=COL6A1 PE=1 SV=1 - [E1BI98_BOVIN]	5.55	6	6	7	0.665	2.209	1.254
E1BB91	Uncharacterized protein OS=Bos taurus GN=COL6A3 PE=1 SV=1 - [E1BB91_BOVIN]	1.89	5	5	5	0.873	1.777	1.433
F1N076	Uncharacterized protein OS=Bos taurus GN=CP PE=1 SV=2 - [F1N076_BOVIN]	35.21	36	36	95	0.686	**2.247**	1.242
F1ML89	Uncharacterized protein OS=Bos taurus GN=CPS1 PE=1 SV=2 - [F1ML89_BOVIN]	1.13	2	2	2	0.741	2.192	1.351
F6R3I5	Uncharacterized protein OS=Bos taurus GN=CRISP3 PE=3 SV=1 - [F6R3I5_BOVIN]	30.17	8	8	15	0.637	2.131	1.110
F1MRU4	Uncharacterized protein OS=Bos taurus GN=DNAH3 PE=4 SV=2 - [F1MRU4_BOVIN]	0.93	2	2	2	0.549	1.714	1.126
E1BFN6	Uncharacterized protein OS=Bos taurus GN=DPYS PE=1 SV=2 - [E1BFN6_BOVIN]	29.46	14	14	14	0.854	2.591	1.429

E1BKT9	Uncharacterized protein OS=Bos taurus GN=DSP PE=1 SV=1 - [E1BKT9_BOVIN]	1.80	6	6	6	0.306	1.053	1.031
G3MZJ0	Uncharacterized protein OS=Bos taurus GN=EIF4EBP3 PE=4 SV=1 - [G3MZJ0_BOVIN]	0.28	2	2	2	0.501	1.583	1.117
F1N6Y7	Uncharacterized protein OS=Bos taurus GN=EIF5B PE=4 SV=2 - [F1N6Y7_BOVIN]	1.23	2	2	2	0.538	1.421	0.997
F1MHP5	Uncharacterized protein OS=Bos taurus GN=FLT4 PE=3 SV=2 - [F1MHP5_BOVIN]	1.77	2	2	3	0.771	3.404	1.555
E1BLA8	Uncharacterized protein OS=Bos taurus GN=GOLM1 PE=4 SV=1 - [E1BLA8_BOVIN]	15.06	6	6	8	0.817	1.934	1.179
E1BB32	Uncharacterized protein OS=Bos taurus GN=GP1BA PE=4 SV=2 - [E1BB32_BOVIN]	15.69	9	9	16	0.749	2.012	1.112
F1N726	Uncharacterized protein OS=Bos taurus GN=GP2 PE=4 SV=1 - [F1N726_BOVIN]	13.67	8	8	8	0.711	1.944	1.179
F1MXE0	Uncharacterized protein OS=Bos taurus GN=HEG1 PE=4 SV=2 - [F1MXE0_BOVIN]	3.19	4	4	4	0.891	1.817	0.966
F1MI25	Uncharacterized protein OS=Bos taurus GN=HIVEP1 PE=4 SV=2 - [F1MI25_BOVIN]	0.45	2	2	2	0.805	1.081	1.204
F1MER7	Uncharacterized protein OS=Bos taurus GN=HSPG2 PE=4 SV=1 - [F1MER7_BOVIN]	0.69	3	3	3	0.900	3.122	1.160
E1BCU8	Uncharacterized protein OS=Bos taurus GN=ICOSLG PE=4 SV=1 - [E1BCU8_BOVIN]	6.56	2	2	3	0.484	1.781	0.904
G3N342	Uncharacterized protein OS=Bos taurus GN=IGHE PE=4 SV=1 - [G3N342_BOVIN]	9.62	5	5	5	0.666	1.923	1.030
G3N2D7	Uncharacterized protein OS=Bos taurus GN=IGLL1 PE=4 SV=1 - [G3N2D7_BOVIN]	51.72	3	3	6	0.850	1.538	1.060
F1MXJ6	Uncharacterized protein OS=Bos taurus GN=KALRN PE=4 SV=2 - [F1MXJ6_BOVIN]	1.19	2	2	2	0.650	2.430	1.410
G3N0V2	Uncharacterized protein OS=Bos taurus GN=KRT1 PE=1 SV=1 - [G3N0V2_BOVIN]	11.06	4	9	18	0.496	1.140	1.124
E1B991	Uncharacterized protein OS=Bos taurus GN=KRT2 PE=1 SV=2 - [E1B991_BOVIN]	8.75	2	7	9	0.832	1.307	1.157
F1MUY2	Uncharacterized protein OS=Bos taurus GN=KRT6B PE=3 SV=1 - [F1MUY2_BOVIN]	37.30	3	23	33	0.352	1.669	1.057
G3MXJ5	Uncharacterized protein OS=Bos taurus GN=LAMP2 PE=1 SV=1 - [G3MXJ5_BOVIN]	7.13	3	3	3	0.681	1.701	1.037
F1MS23	Uncharacterized protein OS=Bos taurus GN=LCN1 PE=4 SV=2 - [F1MS23_BOVIN]	44.38	6	6	6	0.804	1.541	1.132
E1BH06	Uncharacterized protein OS=Bos taurus GN=LOC100852118 PE=1 SV=2 - [E1BH06_BOVIN]	13.79	11	22	24	0.578	2.098	1.137
F1MNJ9	Uncharacterized protein OS=Bos taurus GN=LOC535280 PE=4 SV=2 - [F1MNJ9_BOVIN]	7.95	2	2	2	1.124	4.075	1.515
F1MCF1	Uncharacterized protein OS=Bos taurus GN=LOC782688 PE=3 SV=1 - [F1MCF1_BOVIN]	6.69	2	2	2	2.181	2.910	1.062
A0A0A0MPA0	Uncharacterized protein OS=Bos taurus GN=LOC784932 PE=3 SV=1 - [A0A0A0MPA0_BOVIN]	44.12	11	24	350	0.790	1.954	1.117
F1MC59	Uncharacterized protein OS=Bos taurus GN=LOC787241 PE=3 SV=1 - [F1MC59_BOVIN]	14.58	2	2	2	0.685	1.932	2.073
E1BGJ0	Uncharacterized protein OS=Bos taurus GN=LRP1 PE=4 SV=2 - [E1BGJ0_BOVIN]	2.02	6	6	6	0.841	1.977	1.049
E1B933	Uncharacterized protein OS=Bos taurus GN=LRRIQ1 PE=4 SV=2 - [E1B933_BOVIN]	1.44	2	2	2	0.920	3.282	1.052
F1MVS9	Uncharacterized protein OS=Bos taurus GN=MASP1 PE=3 SV=1 - [F1MVS9_BOVIN]	3.02	2	2	3	1.062	2.342	1.446
F1MET4	Uncharacterized protein OS=Bos taurus GN=MEGF10 PE=4 SV=2 - [F1MET4_BOVIN]	6.25	2	2	2	1.000	1.000	1.000
G3MY87	Uncharacterized protein OS=Bos taurus GN=MGAM PE=3 SV=1 - [G3MY87_BOVIN]	41.16	64	65	197	0.718	**2.037**	1.197
F1MPD1	Uncharacterized protein OS=Bos taurus GN=MRC2 PE=4 SV=1 - [F1MPD1_BOVIN]	1.18	2	2	3	0.800	2.149	1.205
F1MQ37	Uncharacterized protein OS=Bos taurus GN=MYH9 PE=1 SV=2 - [F1MQ37_BOVIN]	1.48	2	2	2	0.376	2.006	0.782
E1BLM2	Uncharacterized protein OS=Bos taurus GN=NRCAM PE=4 SV=2 - [E1BLM2_BOVIN]	3.06	3	3	3	0.793	2.016	1.222
F1MGK8	Uncharacterized protein OS=Bos taurus GN=OSMR PE=4 SV=2 - [F1MGK8_BOVIN]	14.43	4	11	18	0.847	2.399	1.233
F6Q234	Uncharacterized protein OS=Bos taurus GN=PEPD PE=3 SV=1 - [F6Q234_BOVIN]	33.67	15	15	19	0.818	2.371	1.253
E1BH94	Uncharacterized protein OS=Bos taurus GN=PGLYRP2 PE=4 SV=2 - [E1BH94_BOVIN]	12.59	5	5	5	0.756	2.811	1.242
E1BF59	Uncharacterized protein OS=Bos taurus GN=PLEC PE=1 SV=2 - [E1BF59_BOVIN]	0.30	2	2	2	0.504	1.913	0.864
F1MFJ3	Uncharacterized protein OS=Bos taurus GN=PTPRG PE=4 SV=2 - [F1MFJ3_BOVIN]	4.12	5	5	5	0.820	2.019	1.088
F1MGK5	Uncharacterized protein OS=Bos taurus GN=PTPRZ1 PE=4 SV=2 - [F1MGK5_BOVIN]	0.69	2	2	2	0.414	1.811	1.811

G3N2L2	Uncharacterized protein OS=Bos taurus GN=RCN1 PE=4 SV=1 - [G3N2L2_BOVIN]	9.35	2	2	2	0.128	2.764	1.395
E1BNR9	Uncharacterized protein OS=Bos taurus GN=SEMA7A PE=4 SV=1 - [E1BNR9_BOVIN]	8.71	5	5	6	0.711	2.553	1.137
E1BMJ0	Uncharacterized protein OS=Bos taurus GN=SERPING1 PE=3 SV=2 - [E1BMJ0_BOVIN]	6.84	3	3	3	0.628	2.987	1.153
G3MWJ1	Uncharacterized protein OS=Bos taurus GN=SFR1 PE=4 SV=1 - [G3MWJ1_BOVIN]	16.46	2	2	2	0.674	2.126	1.169
F1N2B5	Uncharacterized protein OS=Bos taurus GN=SLC3A2 PE=4 SV=2 - [F1N2B5_BOVIN]	6.73	3	3	4	0.697	2.095	1.181
F1MF78	Uncharacterized protein OS=Bos taurus GN=SYNE2 PE=4 SV=2 - [F1MF78_BOVIN]	0.48	3	3	3	**3.953**	**2.355**	1.068
E1BIG6	Uncharacterized protein OS=Bos taurus GN=TFRC PE=4 SV=2 - [E1BIG6_BOVIN]	9.36	8	8	8	0.732	2.043	1.290
E1BKJ5	Uncharacterized protein OS=Bos taurus GN=TRRAP PE=3 SV=2 - [E1BKJ5_BOVIN]	0.57	2	2	2	0.803	1.826	1.324
Q9GKR3	Uncharacterized protein OS=Bos taurus GN=VCAM1 PE=2 SV=1 - [Q9GKR3_BOVIN]	9.88	6	6	6	0.830	2.014	1.490
F1N789	Uncharacterized protein OS=Bos taurus GN=VCL PE=1 SV=1 - [F1N789_BOVIN]	2.91	4	4	4	0.973	2.412	1.051
Q3ZBS7	Uncharacterized protein OS=Bos taurus GN=VTN PE=2 SV=1 - [Q3ZBS7_BOVIN]	12.61	4	4	5	0.856	3.206	1.272
E1B735	Uncharacterized protein OS=Bos taurus GN=XPNPEP2 PE=3 SV=1 - [E1B735_BOVIN]	6.54	3	3	3	0.726	2.169	1.152
G3N0V0	Uncharacterized protein OS=Bos taurus PE=1 SV=1 - [G3N0V0_BOVIN]	38.34	12	12	29	0.697	2.180	1.158
G5E513	Uncharacterized protein OS=Bos taurus PE=1 SV=1 - [G5E513_BOVIN]	52.95	9	16	70	0.682	2.133	1.229
G5E5T5	Uncharacterized protein OS=Bos taurus PE=1 SV=1 - [G5E5T5_BOVIN]	57.07	6	13	60	0.726	**2.246**	1.150
G5E604	Uncharacterized protein OS=Bos taurus PE=1 SV=1 - [G5E604_BOVIN]	58.88	3	5	18	0.629	1.181	1.000
F1MD73	Uncharacterized protein OS=Bos taurus PE=1 SV=2 - [F1MD73_BOVIN]	9.82	4	4	5	0.822	1.894	1.120
F1MH40	Uncharacterized protein OS=Bos taurus PE=1 SV=2 - [F1MH40_BOVIN]	54.17	3	11	27	0.836	1.740	1.003
A0A0N4STN1	Uncharacterized protein OS=Bos taurus PE=3 SV=1 - [A0A0N4STN1_BOVIN]	18.06	2	2	3	0.826	1.509	1.040
F1N614	Uncharacterized protein OS=Bos taurus PE=3 SV=2 - [F1N614_BOVIN]	4.67	3	3	3	0.609	3.590	1.164
G3MXG6	Uncharacterized protein OS=Bos taurus PE=4 SV=1 - [G3MXG6_BOVIN]	34.53	2	2	3	0.750	2.781	1.312
G3N1H5	Uncharacterized protein OS=Bos taurus PE=4 SV=1 - [G3N1H5_BOVIN]	32.58	2	2	3	0.666	2.802	1.085
G3X7I5	Uncharacterized protein OS=Bos taurus PE=4 SV=1 - [G3X7I5_BOVIN]	37.61	2	2	2	0.466	2.040	1.206
G8JL04	Uncharacterized protein OS=Bos taurus PE=4 SV=1 - [G8JL04_BOVIN]	70.00	4	4	6	1.023	1.752	1.071
E1B9K1	Uncharacterized protein OS=Bos taurus PE=4 SV=2 - [E1B9K1_BOVIN]	32.79	2	2	3	0.920	1.498	0.930
F1MJK3	Uncharacterized protein OS=Bos taurus PE=4 SV=2 - [F1MJK3_BOVIN]	31.49	43	43	88	0.815	**3.338**	**2.175**
F1MLW8	Uncharacterized protein OS=Bos taurus PE=4 SV=2 - [F1MLW8_BOVIN]	32.62	4	8	20	0.711	1.727	1.173
F1MPF3	Uncharacterized protein OS=Bos taurus PE=4 SV=2 - [F1MPF3_BOVIN]	1.68	2	2	2	0.802	2.107	1.074
F1N3L5	Uncharacterized protein OS=Bos taurus PE=4 SV=2 - [F1N3L5_BOVIN]	6.35	5	5	5	0.887	2.490	1.434
F1N757	Uncharacterized protein OS=Bos taurus PE=4 SV=2 - [F1N757_BOVIN]	0.09	2	3	4	1.856	2.095	1.060
P48616	Vimentin OS=Bos taurus GN=VIM PE=1 SV=3 - [VIME_BOVIN]	12.66	5	6	7	**4.065**	**6.724**	**2.217**
F1N5M2	Vitamin D-binding protein OS=Bos taurus GN=GC PE=1 SV=2 - [F1N5M2_BOVIN]	76.79	2	44	362	0.625	**2.112**	1.221
Q3MHN5	Vitamin D-binding protein OS=Bos taurus GN=GC PE=2 SV=1 - [VTDB_BOVIN]	74.68	2	44	349	0.662	**2.167**	1.181
A0A140T851	Vitamin K-dependent protein C OS=Bos taurus GN=PROC PE=3 SV=1 - [A0A140T851_BOVIN]	16.46	6	6	6	0.810	3.002	1.615
P07224	Vitamin K-dependent protein S OS=Bos taurus GN=PROS1 PE=1 SV=1 - [PROS_BOVIN]	5.19	3	3	3	0.903	2.032	1.456
Q3ZCH5	Zinc-alpha-2-glycoprotein OS=Bos taurus GN=AZGP1 PE=2 SV=1 - [ZA2G_BOVIN]	41.14	13	13	43	0.831	1.820	1.094

Supplementary Table 4. Details of specific peptides from proteins detected at a higher level across all OP9-conditioned media.

		Primary media	Secondary media	Tertiary media
		OP9/Ctrl	OP9/Ctrl	OP9/Ctrl
**NPC2**	**Mouse specific peptides**			
	eVNVSPcPTDPcQLHk	42.685	71.498	75.623
	**Other peptides**			
	sGINcPIQk (common to human + mouse)	37.37	50.847	26.613
	nEYPSIk (common to bovine + mouse)	9.744	38.75	12.274
**Follistatin-related protein 1**	**Mouse specific peptides**			
	hQGTAEk	56.706	23.915	17.235
	**Peptide common to Bovine and human**			
	hQETAEk	0.659	2.027	1.087
**Angiotensinogen**	**Mouse specific peptides**			
	gSYNLQDLLAEDk	4.403	4.857	0.775
	nDLLTWIENPPPR	9.793	11.965	9.243
	sLDLSTDPVLATEk	28.323	80.424	74.108
		17.409	100	52.825
	sTcAQLENPSVETLPESTFEPVPIQAk	8.316	35.057	10.642
	tLHDQLVLAAEk	1.753	3.542	2.635
	Median ration from PD	9.024	20.481	9.918
	**Bovine specific peptides**			
	lQAFLGVPGEGQGcTSR	1.086	2.312	1.805
	sLDLSTDPNLAAEk	1.176	2.441	3.066
	sSAVDEEALWEQLVR	1.056	1.657	2.906
		0.643	1.271	2.304
	Median ration from PD	1.071	1.958	2.588
**SPARC**	**Mouse specific peptides**			
	yIAPcLDSELTEFPLR	35.333	83.561	56.506
		100	100	100
		5.512	13.598	6.605
	yIALEEWAGcFGIk	31.49	68.651	29.777
		100	97.675	58.393
	dEGNNLLTEk	90.271	100	24.388
		2.768	3.483	3.228
	Median	35.333	83.561	29.777
	**Bovine specific peptides**			
	yIPPcLDSELTEFPLR	0.788	1.74	0.879
		0.576	1.82	0.961
	yIALDEWAGcFGIk	0.576	1.904	1.038
	dEDNNLLTEk	0.55	1.639	1.101
	Median	0.576	1.78	0.9995
**Collagen alpha-2(I) chain**	**Mouse specific peptides**			
	gEAGAAGPSGPAGPR	3.94	3.627	2.185
	gPAGPSGPVGk	7.643	11.824	5.595
	Median ration from PD	5.488	6.549	3.497
	**Bovine specific peptides**			
	aGVmGPAGSR	4.735	6.114	0.884
	Median	4.735	6.114	0.884
**Dickkopf-related protein 3**	**Mouse specific peptides**			
	eVEELMEDTQHk*	20.128	55.852	12.038
	sHEcIIDEDcGPTR	21.807	29.315	21.418
	ycQFSSFk	0.993	3.309	2.396
	Median	20.128	29.315	12.038
	**Bovine specific peptides**			
	dQQTLcTR	1.12	1.073	1.008
	eVEELMEDTQYk	0.889	1.347	1.264
	gLLFPVcTPLPVEGELcHDPASR	0.967	1.422	1.033
	Median ration from PD	0.967	1.347	1.033

* Same sequence as human but not detected from human database search				
Cathepsin B	**Mouse specific peptides**			
	eQWSNcPTIGQIR	3.342	5.568	5.194
	gENHcGIESEIVAGIPR	1.029	3.332	1.369
	hEAGDMMGGHAIR	4.024	7.731	5.051
	kLcGTVLGGPk	2.668	7.152	3.715
		17.442	55.503	19.208
	lcGTVLGGPk	6.899	31.381	27.531
	scEAGYSPSYk	24.273	40.836	23.133
	scEAGYSPSYkEDk	2.568	7.402	3.555
	vAFGEDIDLPETFDAR	4.394	8.042	5.581
	Median	4.024	7.731	5.194
	**Bovine specific peptides**			
	dAFAADVVLPESFDAR	0.637	2.279	1.137
	eQWPNcPTIk	0.899	1.618	1.218
		0.82	1.71	1.381
	lcGAILGGPk	0.693	1.569	1.109
		0.657	0.53	1.322
	tcEPGYSPSYkEDk	0.711	1.811	1.041
	Median	0.702	1.664	1.1775
Cathepsin Z	**Mouse specific peptides**			
	gDQLALLGR	18.504	16.291	13.363
	nSWGEPWGEk	3.03	5.932	2.294
	sGQTcYHPIR	4.946	8.107	4.468
	Median	4.946	8.107	4.468
	**Human specific peptide**			
	nVDGVNYASITR	0.178	2.072	1.58
Adiponectin	Mouse specific peptides			
	aVLFTYDQYQEk	4.724	5.699	1.64
		3.759	15.627	27.155
	gETGDVGMTGAEGPR	0.941	2.825	1.792
	iFYNQQNHYDGSTGk	1.538	8.868	2.305
		3.92	23.092	12.84
		1.716	26.664	6.075
	sAFSVGLETR	2.02	16.463	2.559
	vTVPNVPIR	8.497	35.074	22.097
	Median ratio from PD	2.756	16.04	3.943
	**Bovine specific peptides**			
	aLLFTHDQFQDk	0.582	1.738	1.345
		0.523	1.74	1.266
		0.748	2.077	1.559
	iFYNQQNHYDGTTGk	0.942	2.37	1.924
		0.727	2.372	1.17
		0.795	2.221	1.342
	nVDQASGSVLLYLEk	0.701	2.064	1.292
		0.719	2.195	1.139
	qVTVPNVPIR	0.661	3.466	1.299
		0.652	1.185	1.514
	sAFSVGLER	0.684	2.419	0.893
	Median ratio from PD	0.701	2.195	1.299
Collagen alpha-1(I) chain	**Mouse specific peptides**			
	sAGVSVPGPMGPSGPR	4.188	14.192	4.288
	**Bovine specific peptides**			
	dcPNAk	0.625	1.702	0.983
	gEAGPSGPAGPTGAR	4.988	1.416	1.67
	gEGGPQGPR	16.403	1.32	1.646
	gETGPAGPAGPIGPVGAR	13.483	1.15	1
	[illegible]	0.87	1.56	0.973
		0.711	1.706	0.991
		0.827	1.896	0.971
		0.912	1.745	1.55

	vPTDEccPVcPEGQESPTDQETTGVEGPk	1.146	2.019	0.981
		0.849	1.742	1.022
		0.685	1.87	1.198
		0.776	1.725	1.587
	Median	0.8595	1.7155	1.011
**Complement factor D**	**Mouse specific peptides**			
	gDSGSPLVcGDAVEGVVTWGSR	4.169	12.667	7.427
	rPDVLHQLR	2.481	9.552	8
	tYHDGVVTINMMcAESNR	1.002	12.126	2.915
	Median ratio from PD	2.481	12.126	7.427
	**Not found in bovine or human**			
**Superoxide dismutase 3 [Cu-Zn]**	**Mouse specific peptides**			
	eVDAAEMHAIcR	7.041	11.349	12.49
	lAccVVGTSSSAAWESQTk	0.778	3.733	2.644
	Median from PD	2.34	6.509	5.747
	**Not found in bovine or human**			
**IGFBP4**	**Mouse specific peptides**			
	tHEDLFIIPIPNcDR	3.495	23.844	8.206
	**Peptide common to all three species**			
	qcHPALDGQR	1.264	6.314	1.331
**Carbonic anhydrase 3**	**Mouse specific peptides**			
	gGPLSGPYR	1.356	3.517	1.917
